# The superfamily keeps growing: Identification in trypanosomatids of RibJ, the first riboflavin transporter family in protists

**DOI:** 10.1371/journal.pntd.0005513

**Published:** 2017-04-13

**Authors:** Darío E. Balcazar, María Cristina Vanrell, Patricia S. Romano, Claudio A. Pereira, Fernando A. Goldbaum, Hernán R. Bonomi, Carolina Carrillo

**Affiliations:** 1 Laboratorio de Parasitología Molecular y Bioquímica, Instituto de Ciencia y Tecnología Dr. Cesar Milstein, Consejo Nacional de Investigaciones Científicas y Técnicas (CONICET). Saladillo, (C1440FFX) Ciudad Autónoma de Buenos Aires, Argentina; 2 Laboratorio de Biología Celular y Molecular, Instituto de Histología y Embriología (IHEM), Universidad Nacional de Cuyo—Consejo Nacional de Investigaciones Científicas y Técnicas (CONICET). Casilla de correo 56, Ciudad de Mendoza, Argentina; 3 Laboratorio de Parasitología Molecular, Instituto de Investigaciones Médicas A. Lanari, Universidad de Buenos Aires—Consejo Nacional de Investigaciones Científicas y Técnicas (CONICET). Combatientes de Malvinas, (C1427ARO) Ciudad Autónoma de Buenos Aires, Argentina; 4 Fundación Instituto Leloir—Instituto de Investigaciones Bioquímicas Buenos Aires (IIBBA)—Consejo Nacional de Investigaciones Científicas y Técnicas (CONICET). Av. Patricias Argentinas 435, (C1405BWE) Ciudad Autónoma de Buenos Aires, Argentina; Harvard School of Public Health, UNITED STATES

## Abstract

**Background:**

Trypanosomatid parasites represent a major health issue affecting hundreds of million people worldwide, with clinical treatments that are partially effective and/or very toxic. They are responsible for serious human and plant diseases including *Trypanosoma cruzi* (Chagas disease), *Trypanosoma brucei* (Sleeping sickness), *Leishmania* spp. (Leishmaniasis), and *Phytomonas* spp. (phytoparasites). Both, animals and trypanosomatids lack the biosynthetic riboflavin (vitamin B2) pathway, the vital precursor of flavin mononucleotide (FMN) and flavin adenine dinucleotide (FAD) cofactors. While metazoans obtain riboflavin from the diet through RFVT/SLC52 transporters, the riboflavin transport mechanisms in trypanosomatids still remain unknown.

**Methodology/Principal findings:**

Here, we show that riboflavin is imported with high affinity in *Trypanosoma cruzi*, *Trypanosoma brucei*, *Leishmania (Leishmania) mexicana*, *Crithidia fasciculata* and *Phytomonas* Jma using radiolabeled riboflavin transport assays. The vitamin is incorporated through a saturable carrier-mediated process. Effective competitive uptake occurs with riboflavin analogs roseoflavin, lumiflavin and lumichrome, and co-factor derivatives FMN and FAD. Moreover, important biological processes evaluated in *T*. *cruzi* (i.e. proliferation, metacyclogenesis and amastigote replication) are dependent on riboflavin availability. In addition, the riboflavin competitive analogs were found to interfere with parasite physiology on riboflavin-dependent processes. By means of bioinformatics analyses we identified a novel family of riboflavin transporters (RibJ) in trypanosomatids. Two RibJ members, *Tc*RibJ and *Tb*RibJ from *T*. *cruzi* and *T*. *brucei* respectively, were functionally characterized using homologous and/or heterologous expression systems.

**Conclusions/Significance:**

The RibJ family represents the first riboflavin transporters found in protists and the third eukaryotic family known to date. The essentiality of riboflavin for trypanosomatids, and the structural/biochemical differences that RFVT/SLC52 and RibJ present, make the riboflavin transporter -and its downstream metabolism- a potential trypanocidal drug target.

## Author summary

In this work, we show that riboflavin plays a key role in the trypanosomatid life cycles and describe a novel family of riboflavin transporters (RibJ) with uptake function. Despite the vital importance of riboflavin for all living cells, RibJ are the first transporters described in protists. We functionally characterized the *T*. *cruzi* and *T*. *brucei* RibJ members and the effect of riboflavin analogs on parasite physiology. The structural and biochemical differences presented between human transporters and RibJ members make riboflavin transport and downstream metabolism, attractive and potential trypanosomatid targets.

## Introduction

Trypanosomatida (class Kinetoplastea) is a major parasitic lineage which infects a high variety of hosts, with insects being their principal vectors. Some trypanosomatids cause common parasitic diseases in humans including Chagas disease (or American Trypanosomiasis) caused by *Trypanosoma cruzi*, sleeping sickness (or Human African Trypanosomiasis) caused by *Trypanosoma brucei* and different manifestations of leishmaniasis (cutaneous, mucocutaneous and visceral leishmaniasis) caused by *Leishmania* spp., with major health impacts around the world [1–3]. Trypanosomatids undergo complex life cycles, involving proliferative and infective stages and intra- or extracellular cycles [1,4–7]. Current clinical treatments are based on drugs generally effective for early-infections and with many associated toxic side effects. Treatments are usually long-lasting and may be difficult to administer; frequently parasites develop resistance against the drugs [8–10]. Hence, there is a clear need to find new therapies against these diseases.

Riboflavin (vitamin B2) is an essential micronutrient for all living cells. It is the precursor of flavin mononucleotide (FMN) and flavin adenine dinucleotide (FAD), cofactors of numerous flavoenzymes playing a pivotal role in redox centers [11]. Metazoa and some microorganisms lack the biosynthetic pathway for riboflavin, obtaining it from the environment through specific transporters [12–14]. In contrast, plants, fungi and most prokaryotes synthesize riboflavin *de novo* [15]. Noteworthy, some of the prototrophic microorganisms that synthesize this vitamin also present exporting and/or importing mechanisms [16–21]. Strikingly, flavin biosynthesis and transport also play a role in microbial infection processes from pathogens and symbionts, as well as in tumorigenesis in some cancer types [19,22–28].

In trypanosomatids, flavoenzymes play important physiological roles including the trypanothione reductase (a FAD disulphide oxidoreductase), the main component of the antioxidant system in trypanosomatids [29]. Similar to Metazoa, *T*. *cruzi* lacks the enzymes involved in *de novo* riboflavin biosynthesis but its genome codes for the enzymes that convert riboflavin into FMN (riboflavin kinase, EC 2.7.1.26) and the latter into FAD (FAD synthetase, EC 2.7.7.2) [30]. This seems to be a general rule for trypanosomatids, but the mechanisms they use to acquire flavins remain to be elucidated. We hypothesized that trypanosomatids require at least one specialized transporter system to import riboflavin from their extracellular environment. In the present work, we studied the role of riboflavin and its uptake in trypanosomatids. This led us to identify and characterize a novel riboflavin transporter family in trypanosomatids, which we named RibJ. Our results provide strong support for the notion that flavin transport and metabolism may be effectively targeted by new therapeutics to be developed against trypanosomiasis.

## Materials and methods

### Parasites and culture media

*Trypanosoma cruzi* epimastigotes of the Y strain (DTU II) expressing GFP (Y-GFP, resistant to G418) [31] and MJ Levin strain (DTU I) were cultivated at 28°C in BHT medium [32], supplemented with 100 U/mL penicillin, 100 μg/mL streptomycin, 10% (v/v) heat-inactivated fetal bovine serum (Natocor) and 20 μg/mL hemin (Sigma). The MJ Levin transgenic cell line was cultivated in medium supplemented with 250 μg/mL G418 (Sigma) for selection. Cell lines expressing GFP did not show differences with respect to the wild-type cells. *Leishmania (Leishmania) mexicana* (Costa Rica strain) promastigotes, *Crithidia fasciculata* (ATCC 11745) choanomastigotes and *Phytomonas* Jma promastigotes were cultured in BHT medium, as described for *T*. *cruzi*. *Trypanosoma brucei* (29–13 strain) procyclic forms were maintained in minimal media consisting on Eagle's minimum essential medium with L-glutamate (US Biological, M3859) supplemented with 0.1 mM L-alanine, 0.1 mM L-asparagine, 0.1 mM L-aspartate, 0.1 mM L-glycine, 0.1 mM L-serine, 2 mM L-glutamine and 5 mM L-proline, 30 mM Na-Hepes (pH 7.3), 26 mM NaHCO_3_, 2 mM sodium citrate, 27 mM glucose, with antibiotics and serum similar to *T*. *cruzi* and 7.5 μg/mL hemin [33].

All cultures were maintained by periodically diluting (each 6–7 days) in fresh medium. When indicated, parasites were cultured in the semi-defined medium SDM-79 [34] or in a modified semi-defined medium with low riboflavin concentration (20 nM), named SDM-20, both supplemented with antibiotics, 10% serum, 7.5 μg/mL hemin and 1 mM putrescine.

### Plasmid constructions

Genomic DNA was extracted from *T*. *cruzi*, *T*. *brucei* or *L*. *(L*.*) mexicana* with UltraPure Phenol (Invitrogen) according to the manufacturer instructions.

A fragment corresponding to *Tc*RibJ (TcCLB.509885.70) was amplified using *T*. *cruzi* genomic DNA as template, primers F-EcoRI-*Tc*RibJ/R-HindIII-*Tc*RibJ and platinum Taq DNA polymerase (Invitrogen). The PCR product was purified, digested with EcoRI and HindIII (New England Biolabs) and ligated using T4 DNA ligase (Invitrogen) into the *T*. *cruzi* expression vector pRIBOTEX [35] to yield pRIBOTEX-*Tc*RibJ.

For heterologous complementation assays in *E*. *coli*, the recombinant expression vectors pET24a*-Tc*RibJ, pET24a-*Tb*RibJ and pET24a-*Lmi*RibJ were constructed harbouring *Tc*RibJ, *Tb*RibJ (Tb927.5.470) and *Lmi*RibJ (LmxM.08_29.2550) fragments from *T*. *cruzi*, *T*. *brucei* and *L*. *(L*.*) mexicana*, respectively. A control vector was constructed using a non-related *T*. *cruzi* permease, *Tc*PAT12 [36,37]. All fragments were amplified from their respective genomic DNA using high-fidelity PCR platinum Pfx DNA polymerase (Invitrogen) and specific primers: F-*Tc*RibJ-NdeI-6xHis/R-*Tc*RibJ-BamHI for *T*. *cruzi*, F-*Tb*RibJ-NdeI-6xHis/R-*Tb*RibJ-BamHI for *T*. *brucei* and F-*Lmi*RibJ-NdeI-6xHis/R-*Lmi*RibJ-BamHI for *L*. *(L*.*) mexicana*. The products were cloned by PCR cloning technique using Q5 high-fidelity DNA polymerase (New England Biolabs) and pET24a plasmid (Novagen) as template, according to the manufacturer instructions.

All constructions were corroborated by sequencing. Primers used in this study are listed in **S1 Table**.

### Parasite transfection

*T*. *cruzi* MJ Levin strain was transfected with pRIBOTEX-*Tc*RibJ and pRIBOTEX-GFP as follows: 10^8^ epimastigote cells grown in BHT medium were harvested by centrifugation, washed with PBS, and resuspended in 0.35 mL of electroporation buffer (PBS containing 0.5 mM MgCl_2_ and 0.1 mM CaCl_2_). The cell suspension was mixed with 50 μg of plasmid DNA in 0.2 cm gap cuvettes (Bio-Rad Laboratories). The parasites were electroporated using a single pulse of (400 V, 500 μF), showing a time constant of ~5 ms. Transfected parasites were cultured in fresh BHT for 24 h and later G418 was added at 250 μg/mL. The MJ Levin strain has been selected for these experiments because of its higher success rate in obtaining transformed clones compared to the Y strain, in the conditions tested during this work.

### Trypanosomatid proliferation assay

The effect of flavins or their analogs on parasites proliferation was evaluated through growth curves. Stationary phase parasites were inoculated in fresh SDM-79 or SDM-20 media (basal 20 nM riboflavin) supplemented to a defined riboflavin concentration. Initial density for *T*. *cruzi* Y-GFP, MJ Levin-GFP (referenced as wild-type) and MJ Levin-*Tc*RibJ (referenced as *Tc*RibJ) was 10^7^ parasite/mL, while initial density for *T*. *brucei*, *L*. *(L*.*) mexicana*, *C*. *fasciculata* and *Phytomonas* Jma was 10^6^ parasites/mL. Riboflavin, flavin mononucleotide (FMN) and flavin adenine dinucleotide (FAD) were dissolved in water and the analogs lumiflavin, lumichrome and roseoflavin (all from Sigma-Adrich) dissolved in DMSO. Then, flavins and analogs were added to the culture media at the indicated concentrations. Parasites were daily counted using a hemocytometer chamber. Proliferation was calculated as the percentage of parasite counts relative to control condition values (flavin at 20 nM or without analogs) on the fifth day. Day 5 was chosen because it is when all parasite cultures tested reach stationary phase in control conditions (**S1 Fig**).

### Riboflavin transport assay in trypanosomatids

The riboflavin uptake measurements were performed using a radiolabeled [^3^H]-riboflavin (6.2 Ci/mmol) (Movarek Biochemicals Inc.) tracer, adapted from arginine transport assays performed by Canepa *et* al. [38] with slight modifications. Briefly, epimastigotes of *T*. *cruzi*, choanomastigotes of *C. fasciculata* and promastigotes of *L. mexicana* and of *Phytomonas* Jma were cultured in BHT, while *T. brucei* procyclic parasites were cultured in SDM-79, to late logarithmic phase. Then, they were harvested and resuspended in fresh media. The cultures were maintained at 28°C until mid-exponential growth, then parasites were harvested, washed three times with PBS-2% glucose, and resuspended in the same buffer for starvation at 28°C with shaking for 3 h. Then, parasites were collected and resuspended in PBS-2% glucose, at a cell density of 300–400 x 10^6^ parasites/mL (30–40 x 10^6^ parasites/tube), and kept at 37°C for 15 min. The assay started after the addition of the radiolabeled riboflavin solution. Riboflavin final concentrations and time points are indicated in each case. To stop the uptake assay, aliquots (0.1 mL) corresponding to each measured point, were placed in 1 mL of stop solution (ice-cold 500 μM unlabeled riboflavin in PBS). Parasites were collected and washed three times with stop solution. Cell pellets were counted for radioactivity in UltimaGold XR liquid scintillation cocktail (Packard Instrument Co., Meridien CT, USA). The kinetic parameters (V_max_ and apparent K_m_) were determined as described in “Kinetic parameters calculation and statistical analysis” section.

Transport displacement assays were performed at 0.3 μM [^3^H]-riboflavin (concentration near to the apparent K_m_ value for riboflavin transport estimated in this work) adding unlabeled flavins or analogs at 10- or 100-fold concentration excess.

To compare the transport activity between MJ Levin-*Tc*RibJ and MJ Levin-GFP strains, parasites were cultivated in SDM-20 fresh medium.

### Bioinformatics analysis

The sequence of the riboflavin transporter Mch5/YOR306C of *Saccharomyces cerevisiae* [21] was obtained from the Saccharomyces Genome Database (http://www.yeastgenome.org/) and employed in the TriTryp database server (http://tritrypdb.org/tritrypdb/) to find similar proteins from trypanosomatid genomes.

The Mch5p, *Tc*RibJ, *Tb*RibJ and *Lmi*Ribj multiple sequence alignment (MSA) was carried out using the MUSCLE program from the EMBL-EBI server (http://www.ebi.ac.uk/Tools/msa/muscle/). Putative N-glycosylation sites of Mch5p, *Tc*RibJ, *Tb*RibJ and *Lmi*RibJ were found using NetNGlyc 1.0 Server (http://www.cbs.dtu.dk/services/NetNGlyc/).

Putative riboflavin transporters similar to *Tc*RibJ from parasites with complete or partially assembled genome sequence were identified using a BLAST search in NCBI database (http://www.ncbi.nlm.nih.gov/assembly) with the exception of *Bodo saltans* and *Trypanoplasma borreli* draft genomes that have been deposited in the Sanger Institute database (http://www.sanger.ac.uk/resources/downloads/protozoa/). The similarity between *Tc*RibJ and other RibJ was determined using ClustalW program with Geneious version 4.8.4 software (http://www.geneious.com/). This program was also used to predict the putative transmembrane regions in RibJ.

The maximum-likelihood tree was constructed with the MEGA6 free software, using a ClustalW MSA of RibJ from kinetoplastids, using bootstrap support (500 pseudoreplicates) and the Le and Gascuel model [39] without Gamma Distribution as the best evolutionary model selected by the software. The accession numbers of all sequences are listed in **S2** and **S3 Tables**.

The neighbor-joining tree was performed with MEGA6 free software (http://en.bio-soft.net/tree/MEGA.html) according to the following pipeline: MSA of riboflavin transporters with the ClustalW program; protein distance calculation with the JTT matrix and neighbor-joining consensus tree construction with a bootstrap support (1000 pseudoreplicates); all bioinformatics tools were used with default parameters. The tree was constructed using the amino acidic sequences from mammalian (RFVT/SLC52), fungi (Mch5), nematodes (*rft*) and kinetoplastid (RibJ) riboflavin transporter families. The results were visualized using iTOL server (http://itol.embl.de/). The accession numbers of all sequences are provided in **S4 Table**.The pairwise global alignment between *Tc*RibJ and human RFVTs sequences, were performed with the software EMBOSS Needle (http://www.ebi.ac.uk/Tools/psa/emboss_needle/). The accession numbers of all sequences are given in **S5 Table**.

### Complementation growth assay in Δ*ribB E*. *coli*

The Δ*ribB*::cat *E*. *coli* strain (Δ*ribB*), kindly provided by García Angulo et al. [19], was used to functionally characterize RibJ riboflavin transporters. Δ*ribB* is unable to import [17] and synthesize [19] riboflavin, and can only be cultured at high riboflavin concentrations. This strain, also resistant to chloramphenicol, was cultured in LB medium at 37°C in a shaker (250 rpm) in the presence of the antibiotic and with excess of riboflavin (750 μM).

The strain Δ*ribB* was transformed using pET24a-*Tc*RibJ, pET24a-*Tb*RibJ, pET24a-*Lmi*RibJ, pET24d-*Tc*PAT12 or an empty vector (pET24a). As a positive control the pET24a-RibM plasmid was included, which codes for the RibM riboflavin transporter from *Streptomyces davawensis* and was kindly provided to us by Dr. Matthias Mack [18]. Selection was carried out on LB agar plates supplemented with kanamycin (50 μg/mL) and excess of riboflavin.

For heterologous expression assays (in solid and liquid media), the ∆*ribB* + pET24a, ∆*ribB* + pET24a-RibM, ∆*ribB* + pET24a-*Tc*RibJ, ∆*ribB* + pET24a-*Tb*RibJ, ∆*ribB* + pET24a-*Lmi*RibJ or ∆*ribB* + pET24a-*Tc*PAT12 strains were cultured overnight in LB broth with kanamycin (50 μg/mL) and excess of riboflavin at 37°C with shaking until the stationary phase. Two mL from each culture were centrifuged at 5,000 rpm, pellets were washed twice with PBS and resuspended in fresh LB with kanamycin added, but no riboflavin. Ten μL aliquots (OD_600_ = 0.01) were used to inoculate LB-agar plates or 2 mL of liquid LB, in both cases supplemented with kanamycin (50 μg/mL), IPTG (0.1 mM) and the indicated riboflavin concentrations for each assay. Cultures were incubated at 37°C for 24 h. Plate images were taken and liquid LB medium cultures OD_600_ values were recorded.

### Riboflavin transport assay in Δ*ribB E*. *coli*

The ∆*ribB* + pET24a, ∆*ribB* + pET24a-RibM, ∆*ribB* + pET24a-*Tc*RibJ and ∆*ribB* + pET24a-*Tb*RibJ strains were cultured overnight in LB medium with kanamycin (50 μg/mL) and excess of riboflavin. Fresh LB medium (kanamycin 50 μg/mL, IPTG 0.4 mM) was inoculated with these bacteria at an initial OD_600_ = 0.01, and incubated at 28°C in the absence of riboflavin, to deplete the intracellular vitamin, until they reached the mid-exponential growth phase (OD_600_ = 0.8–1.2). Cells were harvested, washed three times with PBS- 2% glucose (transport buffer), and resuspended at a final OD_600_ = 5 in the same buffer. The cell suspension was pre-incubated in transport buffer for 15 min at 37°C and the uptake assay started when the [^3^H]-riboflavin solution was added (final concentration 2 μM). Aliquots of 0.2 mL were taken at indicated times and placed in 1 mL of stop solution (ice-cold 500 μM nonradioactive riboflavin in PBS) [19]. Cells were centrifuged at 13,000 rpm for 1 min and washed three times with stop solution. Bacterial pellets were counted for radioactivity in UltimaGold XR liquid scintillation cocktail.

The displacement assay was performed using the same strains in the presence of 0.3 μM radiolabeled riboflavin and the nonradioactive competitors at 3 μM and 30 μM.

In all cases, the value obtained with ∆*ribB* + pET24a, which corresponds to unspecific binding, was subtracted from the other measurements.

### Antibiotics susceptibility assay

This assay was performed to corroborate that the permeability of ∆*ribB E*. *coli* was not affected by the expression of heterologous riboflavin transporters. Transformed *E*. *coli* ∆*ribB* strains were cultivated in liquid LB with excess of riboflavin (750 μM) and bactericidal compounds (0–40 μg/mL nalidixic acid or 0–250 μg/mL acriflavine), then growth IC_50_ values were determined.

### *T*. *cruzi in vitro* metacyclogenesis assay

The *in vitro* differentiation assay was performed as previously described [40]. Briefly, 7-day-old *T*. *cruzi* epimastigotes (Y-GFP strain) cultured in SDM-20 were harvested and incubated for 2 h at 37°C in triatomine artificial urine (TAU) medium. Next, parasites were diluted in TAU3AAG medium (TAU supplemented with 10 mM L-proline, 50 mM L-sodium glutamate, 2 mM L- sodium aspartate and 10 mM D-glucose) with the addition of 0–300 nM riboflavin, FMN or FAD, or 10 μM analogs. Parasites were cultured at 28°C for 48 h. The epimastigotes and differentiated metacyclic trypomastigotes (MT) mix was harvested by centrifugation at 600 × g for 15 min and resuspended in 0.5 mL of fresh human serum, which selectively lyses epimastigotes [41]. MT, easily seen by light microscopy, were quantified using a hemocytometer chamber.

### *T*. *cruzi in vitro* infection assay

The *in vitro* infection assay was performed following protocols previously described [40]. Briefly, *T*. *cruzi* tissue culture trypomastigotes (TCT) (Y-GFP strain), obtained from VERO cells, were pre-treated with 10 μM riboflavin analogs at 37°C for 2 h. Subsequently, monolayers of H9C2 cardiomyoblasts, which had been grown in DMEM-10% fetal bovine serum (FBS) in 24-well plates containing glass coverslips, were infected with the TCT using 10 parasites per cell (MOI 10:1). The co-cultures were maintained in the presence of 10 μM riboflavin analogs at 37°C for 12 h allowing the cardiomyoblast infection. Unbound TCTs were removed by washing with fresh DMEM, and infected mammalian monolayers were incubated in DMEM-3% FBS, 10 μM riboflavin analogs, at 37°C for 48 h. For these assays, riboflavin analogs were dissolved in a DMSO/DMEM solution (1:100 v/v). Then, samples were fixed with 10% paraformaldehyde; the actin cytoskeleton of the H9C2 cells was stained with TRITC-phalloidin (Invitrogen) and parasites were directly visualized due to the stable expression of GFP [40]. Finally, cell invasion (expressed as percentage of infected host cell) and amastigotes proliferation (number of amastigotes per cell) were quantified using confocal microscopy (FV1000 Confocal Olympus microscope).

### Kinetic parameters calculation and statistical analysis

Standard procedures were used to determine kinetic parameters. The apparent K_m_ and V_max_ values were obtained by nonlinear regression fit of the Michaelis-Menten equation to the data. Statistics, curve fitting, V_max_ and apparent K_m_ were calculated using the GraphPad Prism 6 software.

Each experiment was carried out at least three times. Groups were analyzed using one-way ANOVA test followed by a post-hoc Tukey's multiple comparison test (significance cut-off value P = 0.05). The infectivity and amastigote replication assays were analyzed using a nonparametric test (Kruskal-Wallis) and compared against the control condition through Dunn’s test (significance cut-off value P = 0.05).

The correlation analysis was performed between the maximum density values reached with riboflavin-supplemented media from each parasite and the apparent K_m_ values for riboflavin uptake. The correlation was estimated using the Pearson coefficient (significance cut-off value P = 0.05, two-tailed). A linear regression was represented with confidence intervals at 95%.

### Ethics statement

All parasites used during this work are laboratory strains. *T*. *cruzi* Y-GFP, *Phytomonas* Jma and *C*. *fasciculata* (ATCC 11745) strains were previously reported by our group [40,42,43]. *T*. *cruzi* MJ Levin strain was provided by Dr. Claudio Pereira [44]. *T*. *brucei* (29–13 strain) and *L*. *(L*.*) mexicana* (Costa Rica strain) were kindly provided by Dr. Guillermo Alonso [45] and Dr. Carlos Labriola [32], respectively.

## Results

### Effects of flavins on proliferation of trypanosomatids

**Fig 1:**
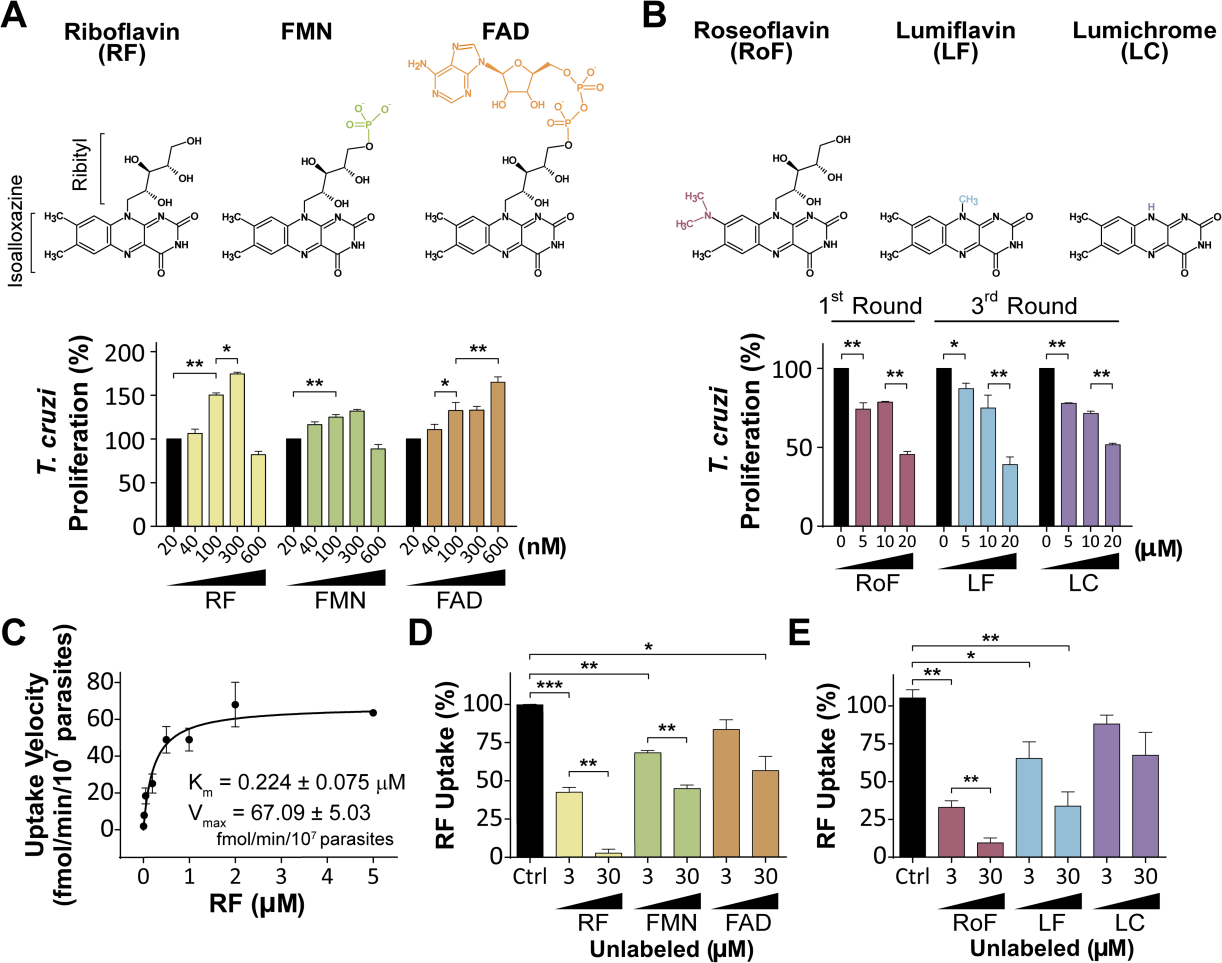
Flavins and chemical analogs are incorporated into *T*. *cruzi* epimastigotes and affect their proliferation with opposite effects. Chemical structures of riboflavin, FMN and FAD, and their analogs roseoflavin, lumiflavin and lumichrome are shown in A and B top panels. A and B bottom panels: *T*. *cruzi* Y strain epimastigotes were maintained at 28°C until stationary phase, then washed and incubated in fresh medium with the indicated compound concentrations: (A) flavins and (B) chemical analogs plus 300 nM riboflavin. Parasites were counted daily. *T*. *cruzi* proliferation (%) was calculated at the indicated round using fifth day-counts and control conditions -(A) 20 nM flavins or (B) 0 μM analogs- as references (100%). Log-phase Y strain epimastigotes grown in BHT-10% FBS were harvested, washed, resuspended in PBS-2% glucose and incubated at 37°C. (C) Riboflavin uptake velocity was calculated at 0–5 μM final substrate concentration. Aliquots were sampled at 0 and 5 min after the addition of radioactive material. Displacement assays were performed at 0.3 μM radioactive riboflavin mix (Ctrl: control, 100%) and 3–30 μM of (D) unlabeled flavins (RF, FMN or FAD) or (E) unlabeled analogs (RoF: roseoflavin, n = 3; LF: lumiflavin, n = 4; or LC: lumichrome; n = 4). Values are expressed as mean ± SD. Statistical analysis was performed by one way ANOVA test followed by a post-hoc Tukey's multiple comparison test (*P < 0.05, **P < 0.01, ***P < 0.005).

As trypanosomatids lack biosynthetic enzymes for B-vitamins, we tested the effects that extracellular flavins exert on proliferation of trypanosomatids. *T*. *cruzi* epimastigotes moderately proliferated at low concentration (20 nM) of riboflavin or its derivatives FMN and FAD. Higher concentrations of flavins (< 300 nM) significantly increased the *T*. *cruzi* proliferation (74.3 ± 3.6% for riboflavin, 32 ± 3.2% for FMN and 32.8 ± 7.7% for FAD, compared to the control); concentrations higher than 300 nM of riboflavin and FMN had a negative proliferation effect (**Fig 1A**). Flavins also showed a stimulatory effect on proliferation of *T*. *brucei* procyclic parasites, *C*. *fasciculata* choanomastigotes and promastigotes of *L*. *(L*.*) mexicana* and *Phytomonas* Jma, exhibiting slight differences in doses-response profiles (**S2 Fig**).

On the other hand, the riboflavin analogs roseoflavin [46], lumiflavin and lumichrome [47] affected trypanosomatid proliferation rates. While lumiflavin and lumichrome (at 10 μM) showed effects on *T*. *cruzi* in the third round of culture (32.8 ± 6.3% and 28.5 ± 2.5% lower than the control, respectively), roseoflavin treatment resulted in a marked reduction of parasite proliferation in the first round of culture (21.4 ± 0.8% lower than control) (**Fig 1B** and **S3 Fig**).

These results suggest that trypanosomatids incorporate extracellular flavins and that one could interfere with flavin uptake and/or the downstream metabolism to affect proliferation of the parasites.

### Riboflavin uptake in trypanosomatids

A [^3^H]-riboflavin transport assay was performed to measure its uptake in trypanosomatids. *T*. *cruzi*, as well as *T*. *brucei*, *L*. *(L*.*) mexicana*, *C*. *fasciculata* and *Phytomonas* Jma showed riboflavin uptake following Michaelis-Menten kinetics, with a maximal velocity at 1–2 μM of riboflavin and apparent K_m_ values in the submicromolar range, indicative for the involvement of a high-affinity transporter (**Fig 1C, S4 Fig** and **Table 1**). Riboflavin derivatives were efficient competitors of the [^3^H]-riboflavin uptake in *T*. *cruzi* epimastigotes, with FMN being more effective than FAD (**Fig 1D**). On the other hand, lumichrome showed mild competitive effects, while roseoflavin and lumiflavin significantly reduced 70 to 90% and 35 to 65% the [^3^H]-riboflavin uptake, respectively (**Fig 1E**).

**Table 1:** Riboflavin apparent K_m_ and V_max_ uptake parameters measured in trypanosomatids.

Trypanosomatid	K_m_ (μM)	V_max_ (fmol/10^7^ parasites · min)
***T*. *cruzi***	0.22 ± 0.08	67.09 ± 5.03
***T*. *brucei***	0.39 ± 0.08	38.00 ± 2.10
***L*. *(L*.*) mexicana***	0.07 ± 0.03	42.80 ± 3.20
***C*. *fasciculata***	0.14 ± 0.04	108.10 ± 6.30
***Phytomonas* Jma**	0.35 ± 0.11	12.20 ± 1.00

### RibJ: A novel family of riboflavin transporters in trypanosomatids

**Fig 2:**
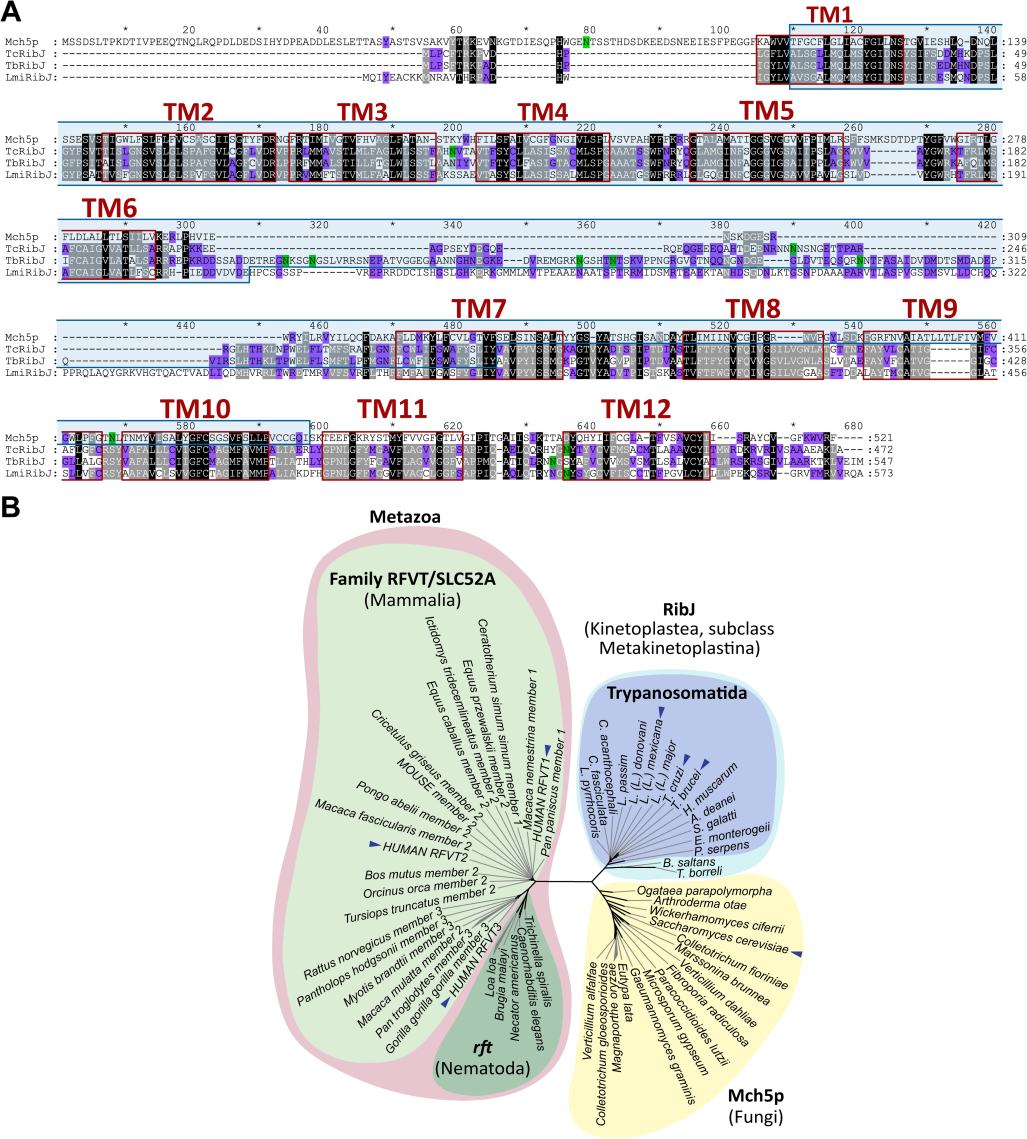
RibJ is a novel family of riboflavin transporters characteristic of trypanosomatids. (A) Multiple sequence alignment between Mch5p (*S*. *cerevisiae*), *Tc*RibJ, *Tb*RibJ and *Lmi*RibJ using MUSCLE program. Red boxes: putative transmembrane domains; light blue box: MFS domain; green boxes: the Asn in N-glycosylation context. Black, gray and violet backgrounds represent 100%, 80%, and 60% conservation within similarity groups, respectively. (B) A Neighbor-joining tree (1000 pseudoreplicates) was constructed using the amino acid sequences from riboflavin transporters: 22 mammalian (RFVT/SLC52, light green), 15 fungal (Mch5p, yellow), 5 nematodal (*rft*, dark green) and 16 RibJ belonging to subclass Metakinetoplastids (light blue, and inside, trypanosomatids group in violet). The blue arrows indicate RibJ from *T*. *cruzi*, *T*. *brucei* and *L*. *(L*.*) mexicana*, and the human and *S*. *cerevisiae* riboflavin transporters.

As a first approach for *in silico* studies, sequences of previously characterized transporters from bacteria, *Saccharomyces cerevisiae*, *Caenorhabditis elegans* and mammals [12–14,16–21] were used as queries in BLAST searches against the *T*. *cruzi* genome. Only the riboflavin transporter Mch5p from *S*. *cerevisiae* showed a hit with low identity (21%) and similarity (37%) values. The gene alleles, TcCLB.509885.70 and TcCLB.508397.70, are encoded in chromosome 28-S and 28-P, respectively. This putative riboflavin transporter, which we named here *Tc*RibJ, is 472 amino acids long and contains three possible N-glycosylation sites (N108, N236 and N431) and 12 hydrophobic regions, probably corresponding to 12 transmembrane segments, which are also present in Mch5p (**Fig 2A**).

The *Tc*RibJ sequence was used as query to find homologs in *T*. *brucei* and *L*. *(L*.*) mexicana*, finding ortholog genes that code for proteins similar to *Tc*RibJ: *Tb*RibJ (62.2% identity) and *Lmi*RibJ (48.9% identity). The three RibJ members showed at the N-terminal region a Major Facilitator Superfamily domain (MFS domains), found in small solute transporters [48] (**Fig 2A** and **S2 Table**).

RibJ orthologs, exhibiting an MFS domain and 12 predicted transmembrane segments, were retrieved from totally or partially assembled genomes from representatives of the Metakinetoplastina subclass (**S2**and **S3 Tables**, respectively). RibJ members were even found in phylogenetically distant taxa from *T*. *cruzi*, as the free-living kinetoplastid *Bodo saltans* (order Eubodonida) and the fish endoparasite *Trypanoplasma borreli* (order Parabodonida), while the *Perkinsela* sp. genome (subclass Prokinetoplastina) did not show any recognizable homolog. Using all these identified sequences, a phylogenetic maximum-likelihood tree was constructed (**S5 Fig**) with a topology in accordance with the currently accepted Kinetoplastea phylogeny [49]. Therefore, it is likely that the RibJ transporter family has a common origin in Metakinetoplastids, which diversified reminiscently of the speciation process, with *Phytomonas* spp. RibJ being the most distant transporter in the order.

To complete the phylogenetic analysis of eukaryotic riboflavin transporters, the amino acid sequences from the human riboflavin transporter (RFVT1/SLC52A1) [12], *C*. *elegans rft-1* [13] and yeast Mch5p [21] were used as queries in a BLAST search in the NCBI database. Representative sequences comprising the mammalian, nematodal and fungal datasets (**S4 Table**) and, including the kinetoplastid RibJ transporters, were used to construct a neighbor-joining tree (**Fig 2B**). Riboflavin transporter members cluster in three distinct major groups, revealing that RibJ constitutes a novel family, the first one identified in protists and the third in eukaryotes, distant from the RFVT/SLC52 family and more related to the Mch5p family (97% bootstrap support).

### Functional characterization of RibJ members

**Fig 3:**
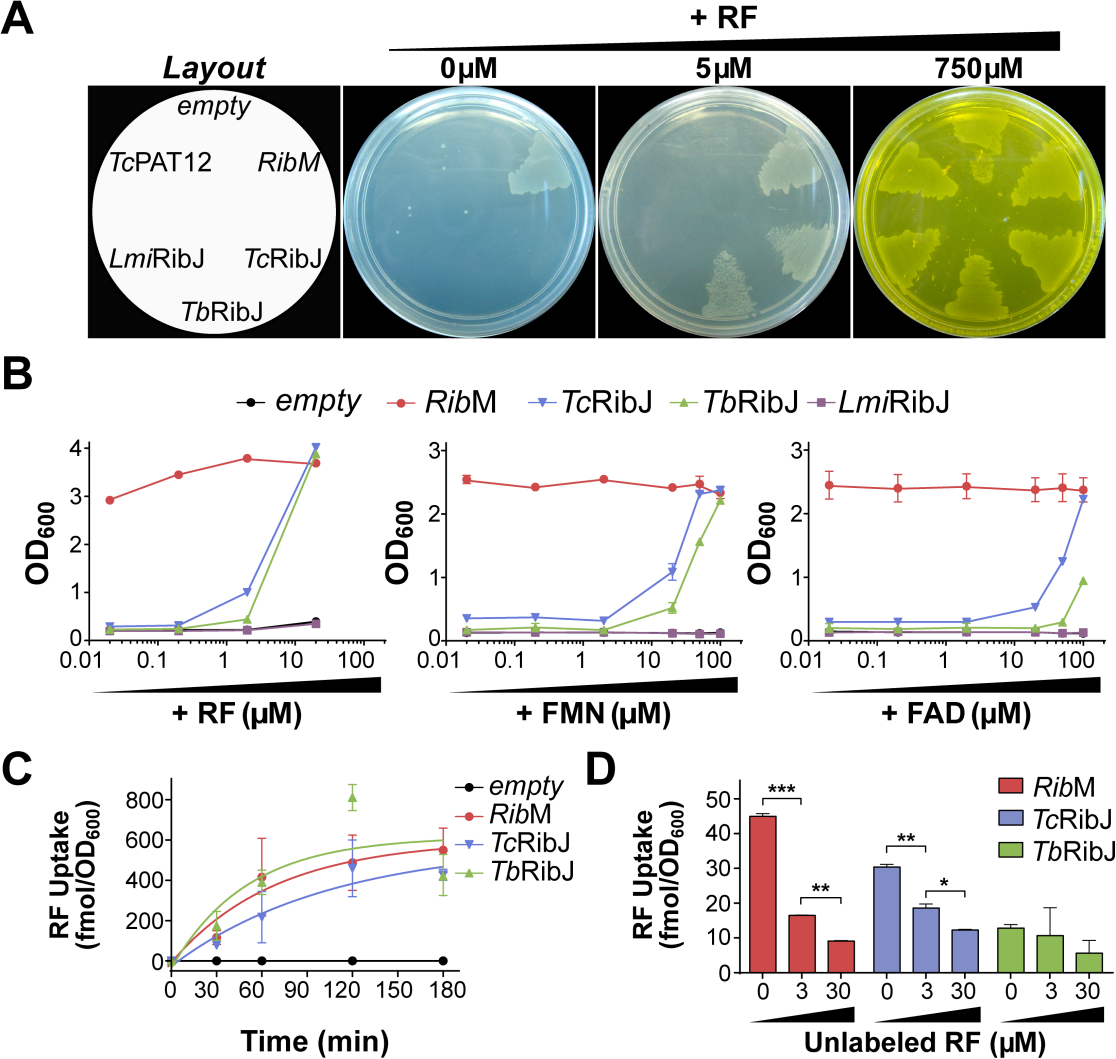
*Tc*RibJ and *Tb*RibJ display flavin transport activity in *E*. *coli*. The *E*. *coli* ∆*ribB* strain was transformed with expression plasmids coding for either RibM, *Tc*RibJ, *Tb*RibJ, *Lmi*RibJ or *Tc*PAT12, or with an empty vector. Strains were (A) plated on LB agar (left: strain plating scheme) with the addition of 0, 5 or 750 μM riboflavin (RF) or (B) cultured at 37°C for 24 h in liquid LB supplemented with 0.02–100 μM FMN or FAD, or 0.02–20 μM riboflavin. (C) [^3^H]-riboflavin uptake (2 μM) was measured from 0 to 180 min in bacteria expressing RibM, *Tc*RibJ or *Tb*RibJ or containing an empty vector. (D) Displacement assays performed with 0.3 μM [^3^H]-riboflavin in the absence of competitors (Ctrl: control) or in the presence of 3–30 μM of unlabelled riboflavin; aliquots were sampled at 0 and 120 min after the addition of the radioactive material. Values are expressed as mean ± SD. Statistical analysis was performed by one way ANOVA test followed by a post-hoc Tukey's multiple comparison test (*P < 0.05, **P < 0.01, ***P < 0.005).

To evaluate the functionality of RibJ as a riboflavin transporter we designed an experiment of heterologous expression to restore growth of a riboflavin auxotrophic bacterium. The *ribB E*. *coli* null mutant strain (∆*ribB*) [19] was transformed with expression vectors carrying either the *Tc*RibJ, *Tb*RibJ or *Lmi*RibJ gene. An empty vector and one harbouring the *Tc*PAT12 gene (the polyamine transporter of *T*. *cruzi*) [36,37] were included as negative controls, and a plasmid encoding the RibM riboflavin transporter from *Streptomyces davawensis* [18] as a positive control. All transformants grew when plated on LB medium plus an excess (750 μM) of riboflavin, while only Δ*ribB* expressing RibM could grow when no riboflavin was supplemented (**Fig 3A**), since RibM incorporates riboflavin traces present in the LB medium [19]. When riboflavin was added in a restrictive concentration (5 μM), *Tc*RibJ and *Tb*RibJ also supported ∆*ribB* growth (**Fig 3A**). Similar results were obtained in liquid LB medium assays, where *Tc*RibJ and *Tb*RibJ restored the growth capacity in the presence of riboflavin, and also FMN and FAD, although at higher concentrations (**Fig 3B**). These findings confirm that RibJ proteins possess flavin transporter activity *in vivo*. In all conditions, negative controls (*Tc*PAT12 or empty vector) failed in restoring Δ*ribB* growth. Strikingly, despite the similarities with the other RibJ, *Lmi*RibJ was unable to transport riboflavin in this heterologous system (**Fig 3A and 3B**).

*Tc*RibJ and *Tb*RibJ heterologous expression allowed Δ*ribB* strains to incorporate [^3^H]-riboflavin with a linear time-dependent velocity for the first 60 min at 4.1 and 7.3 fmol/OD_600_.min, respectively (**Fig 3C**). The transport specificity was confirmed by displacement assays performed at 10- or 100-fold unlabeled riboflavin excess (**Fig 3D**). To rule out membrane permeability alterations or unspecific transport due to the heterologous expression of membrane proteins, an antibiotics susceptibility assay was performed. IC_50_ values for nalidixic acid and acriflavine in *E*. *coli* ∆*ribB* strains (0.47–0.76 μg/mL and 21.3–29.1 μg/mL, respectively) (**S6 Fig**) were similar to those reported in the literature [50,51], indicating that the flavin uptake measured in these strains was the result of specific flavin transporter activity.

### *Tc*RibJ is a flavin transporter in *T*. *cruzi*

**Fig 4:**
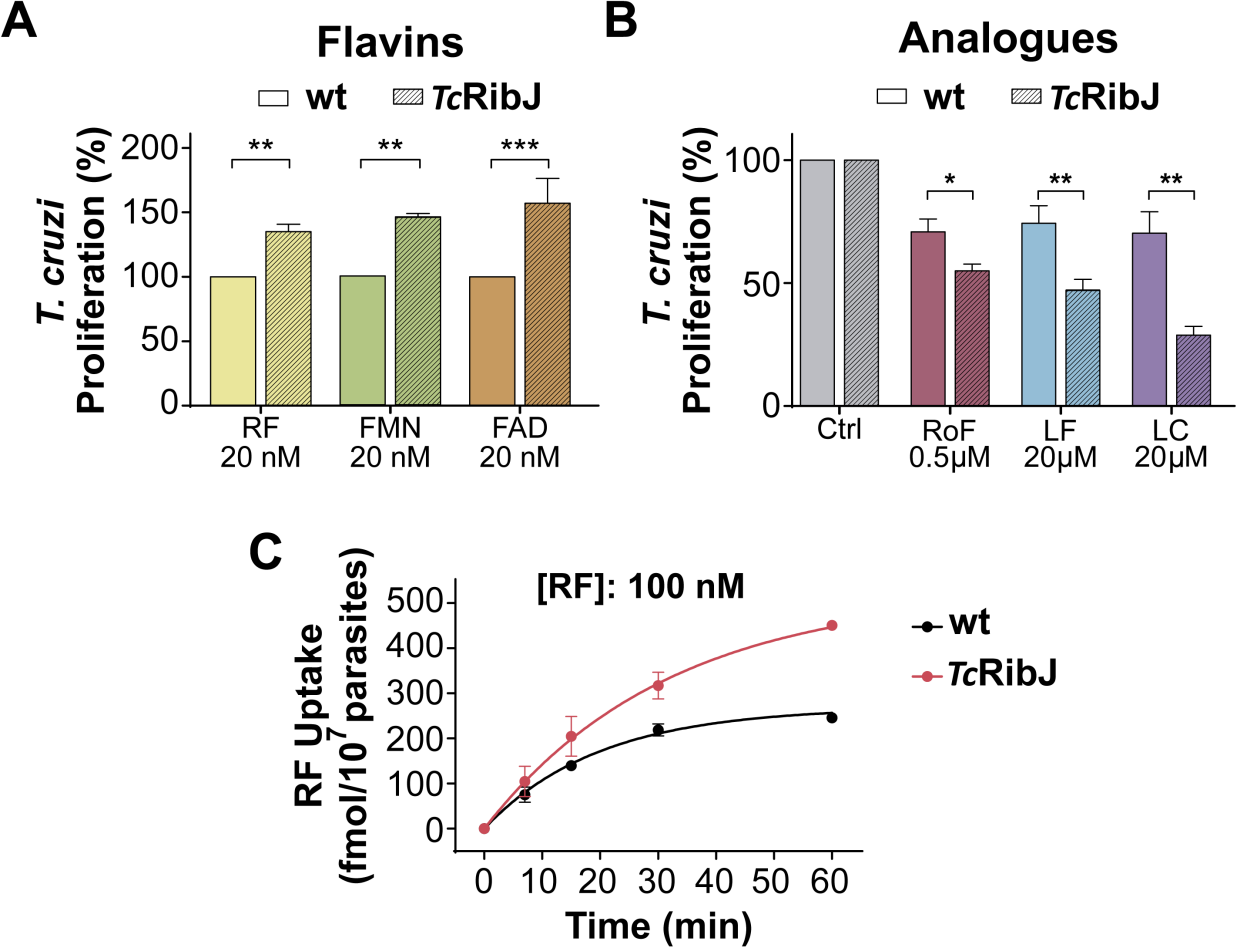
*Tc*RibJ functions as flavin transport *in vivo* in *T*. *cruzi*. Epimastigotes transfected with pRIBOTEX-GFP (wt) or pRIBOTEX-*Tc*RibJ (*Tc*RibJ) were incubated in fresh SDM-20-10% FBS in the presence of (A) 20 nM riboflavin (RF), FMN or FAD, or (B) roseoflavin (RoF), lumiflavin (LF), or lumichrome (LC), at the indicated concentrations. Parasites were counted daily using a hemocytometer chamber. *T*. *cruzi* proliferation (%) was calculated at the third culture round, where the control condition (20 nM flavin) was referenced as 100%. (C) [^3^H]-riboflavin (100 nM) uptake measurements in control and over-expressing *Tc*RibJ epimastigotes. Values are expressed as mean ± SD. (A-B) Statistical analysis was performed by a two-tailed unpaired t test (*P < 0.05, **P < 0.01, ***P < 0.005).

To confirm the RibJ functionality in trypanosomatids, we transformed *T*. *cruzi* epimastigotes with *Tc*RibJ to generate an over-expressing strain (*Tc*RibJ). This strain showed increased proliferation rates in the presence of low concentrations (20 nM) of any flavin (riboflavin, FMN or FAD) (**Fig 4A**), higher sensibility to riboflavin analogs (**Fig 4B**) and increased riboflavin uptake (1.5 ± 0.4-fold) (**Fig 4C**), compared to the wild-type strain. These results confirm that *Tc*RibJ is a functional flavin transporter in *T*. *cruzi*, and that its activity affects proliferation of epimastigotes.

### Flavins play an important role in *T*. *cruzi* life cycle

**Fig 5:**
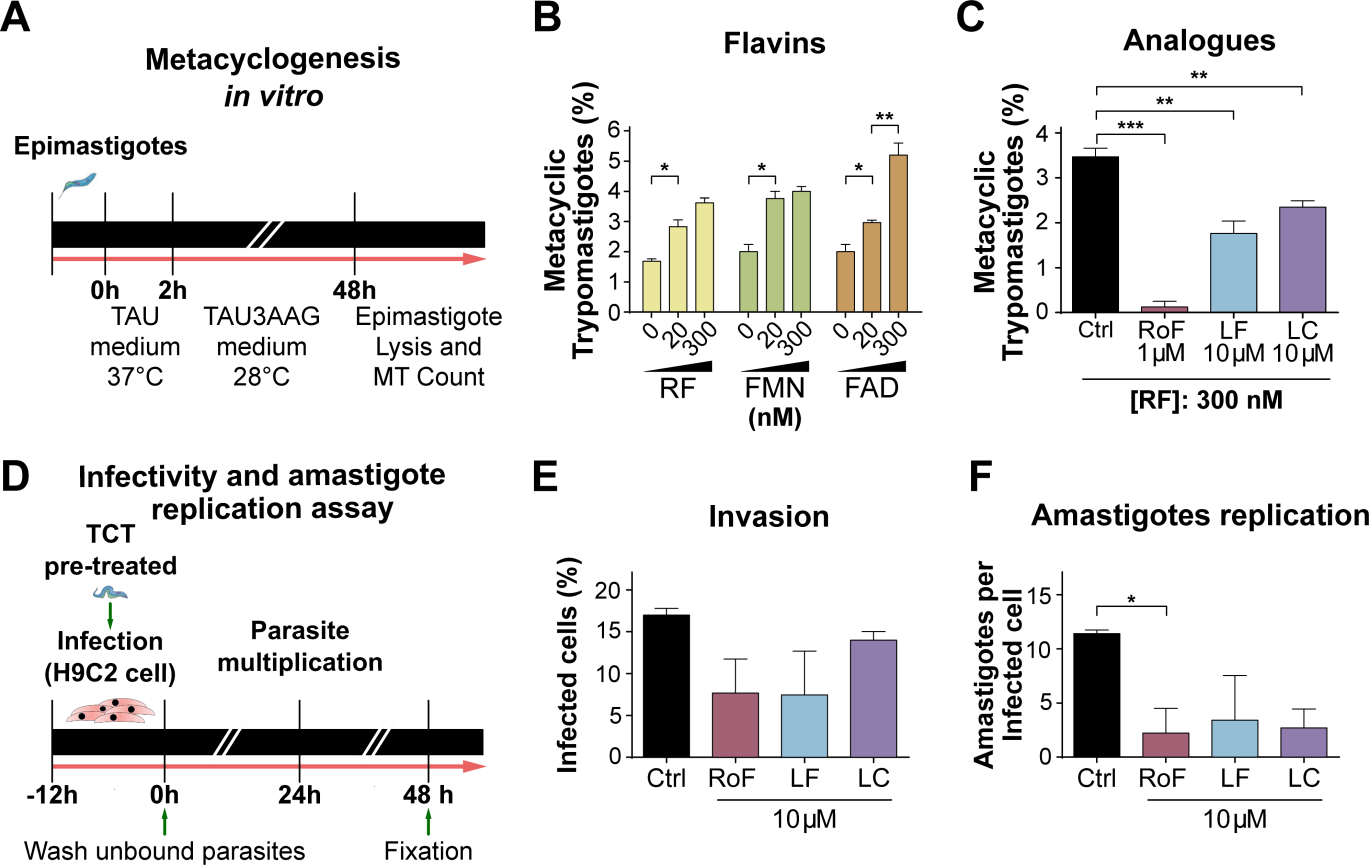
Flavins stimulate while analogs retard progression through *T*. *cruzi* life cycle. (A) *In vitro* metacyclogenesis assay scheme (for more details, see ‘Material and methods‘ section). Percentage of MT obtained in differentiation media (TAU3AAG) supplemented with (B) flavins (riboflavin: RF, FMN or FAD), or (C) chemical analogs (roseoflavin: RoF, lumiflavin: LF, or lumichrome: LC), at the indicated concentrations at 48 h. (D) *In vitro* infection assay scheme (for more details, see ‘Material and methods‘ section). Effect of 10 μM analogs on (E) cellular invasion at 48 h, expressed as percentage of H9C2 host cells infected with *T*. *cruzi* Y-GFP (∼400 cells counted) or (F) amastigote proliferation at 48 h, expressed as number of *T*. *cruzi* Y-GFP amastigotes per infected H9C2 host cell. Values are expressed as mean ± SD. Statistical analysis was performed by a Kruskal-Wallis non-parametric test followed by a post-hoc Dunn's multiple comparison test (*P < 0.05, **P < 0.01, ***P < 0.005).

To get a broader view of the role that flavins play in trypanosomatid physiology, we tested how they affect *T*. *cruzi* metacyclogenesis (**Fig 5A**). When flavins were added to the differentiation medium (TAU3AAG), a significant higher percentage of epimastigotes differentiated to metacyclic trypomastigotes (MT) compared to control conditions, while all flavin analogs produced a dramatic reduction of MT counts (**Fig 5B and 5C**). Strikingly, roseoflavin completely abolished MT counts (**Fig 5C**); however, it still remains to be determined whether this effect is a consequence of affecting (i) the viability of epimastigotes in the process of metacyclogenesis, (ii) directly metacyclogenesis and/or (iii) MT viability.

Once inside the mammalian host, cellular invasion and intracellular replication are essential events for a successful *T*. *cruzi* infection [40]. Hence, we evaluated the effect of flavins on these processes in an *in vitro* infection model (**Fig 5D**). Flavin analogs produced a mild reduction in infected host cell counts (**Fig 5E**) and reduced amastigote intracellular proliferation, with statistically significance for roseoflavin (11.4 ± 0.3 vs 2.2 ± 2.3 amastigotes per infected cell for control and roseoflavin treatment, respectively) (**Fig 5F**).

Taken together, these results strongly suggest that flavins are necessary for metacyclogenesis and amastigote proliferation in *T*. *cruzi*, and that interfering with riboflavin transport and/or downstream metabolism may impair these processes.

## Discussion

To date, only few studies on B-vitamins transporters have been reported for trypanosomatids: (i) folic acid transporters were molecularly characterized in *Leishmania* spp. [52]; (ii) *myo*-inositol transporter genes were identified in *Leishmania* spp. and *T*. *brucei*, while a biochemical characterization was performed of this transporter in *T*. *cruzi* [53]; and (iii) the choline uptake was biochemically studied in *Leishmania* sp. and *T*. *brucei*, but choline transporter genes still remain to be identified [54,55].

Our results demonstrate that extracellular flavins are naturally incorporated in trypanosomatids, affecting their proliferation (**Figs 1** and **S2–S4**). In all trypanosomatids assessed, riboflavin uptake is mediated by high-affinity transporters, presenting K_m_ values in the nanomolar range (**Fig 1**and **Table 1**). In contrast, other microorganisms show low-affinity riboflavin transport, for example, *S*. *cerevisiae* and *Ashbya gossypii* exhibits K_m_ in the micromolar order with values of 17 and 40 μM, respectively [21,56]. High-affinity transporters are commonly found in trypanosomatids and it is assumed as an evolutionary adaptation to their restricted nutritional environments [57]. Their invertebrate vectors obtain riboflavin from the diet and their microbiota, and provide a vitamin restrictive environment for parasites [58,59]. Even more, parasites in mammalian hosts are exposed to very low riboflavin concentration, within the nanomolar range in human plasma [60] and attomolar intracellular concentrations [61]. On the other hand, *Phytomonas* spp. inhabit a millimolar flavin environment once inside their host plants [62], developing a particular energy metabolism that relies on flavoenzymes [63]. In all cases, trypanosomatids seems to be dependent on effective riboflavin uptake.

While the proliferation of *T*. *cruzi* epimastigotes, *T*. *brucei* procyclic forms and *C*. *fasciculata* choanomastigotes was strongly promoted by riboflavin, the proliferation of *L*. *(L*.*) mexicana* and *Phytomonas* Jma promastigotes was slightly stimulated by it (**Figs 1**and **S2**). These differences found on proliferation inversely correlate with their corresponding transport affinity (**S7 Fig**, P < 0.05). Thus, parasites with less efficient riboflavin uptake present higher proliferation when riboflavin is supplemented in the media. Contrarily, the moderate-proliferative parasites upon riboflavin addition exhibit higher uptake affinities. This could suggest that there exists regulatory mechanisms acting on riboflavin transport to prevent flavin accumulation in high levels. In this sense, it was reported that flavin excess inhibit the expression of several genes associated with riboflavin obtaining in bacteria [64,65]; also, regulatory mechanisms by nutrients availability have been reported in several trypanosomatids [66]. Interestingly, *Phytomonas* Jma is out of this correlation (**S7 Fig**), and the reason that may explain this is the high flavin concentrations in its niche [62].

At certain extracellular flavin concentrations, a negative effect on trypanosomatid proliferation is produced (**Figs 1A** and **S2**), as similarly described for vitamins B12 (cobalamin) and B3 (nicotinamide) [67–69]. Previous reports show that an oxidative redox environment promotes the *T*. *cruzi* epimastigotes proliferation by activating the Calcium/calmodulin-dependent protein kinase II (CaMKII) pathway, while in a reductive environment its proliferation is arrested [70,71]. Thus, it is possible that some highly flavin enriched media (e.g. 600 nM riboflavin for *T*. *cruzi*, **Fig 1A**) favors a reductive environment for the parasites, impairing trypanosomatid proliferation. However, we cannot exclude another mechanisms such as direct or indirect effects at gene expression levels produced by riboflavin, as reported in bacteria and mammalian cells [65,72]. Interestingly, **S2 Fig** also shows that high flavin concentration in some cases still produced a positive stimulus on proliferation in *T*. *brucei*, *L*. *(L*.*) mexicana* or *C*. *fasciculata* (eg. 600 nM FAD for *T*. *brucei*, 600 nM riboflavin for *L*. *mexicana* and 600 nM FMN for *C*. *fasciculata*). A possibility is that beyond certain threshold levels, there are onsets of active mechanisms controlling the intracellular flavin concentrations, for example by flavin exporters. The existence of such flavin-exporting activities have already been described for bacteria and mammals [18,73–75]. However, the existence of flavin exporters in trypanosomatids remains unknown to date.

We have identified the novel family of riboflavin transporters RibJ, which is distinct and distant from the two riboflavin transporters families previously described in eukaryotes, and the first riboflavin transporter reported for protists (**Fig 2**). Since trypanosomatid parasites frequently have redundant transport activities to guarantee supply from different nutritional environments, as described for other compounds (amino acids, glucose, etc.) [38,76–80], we cannot exclude the presence of additional yet unidentified riboflavin transporters. *T*. *cruzi* and *T*. *brucei* RibJ members were functionally validated *in vivo* as flavin transporters using a heterologous expression assay (**Fig 3**) and in a homologous over-expression system in *T*. *cruzi* epimastigotes (**Fig 4**). Although *L*. *(L*.*) mexicana* transports riboflavin with the highest affinity compared with the other parasites analyzed in this work, we could not confirm the *Lmi*RibJ functionality by the heterologous complementation assay. One possible explanation is that *Lmj*RibJ presents low or null expression levels or it does not adopt a functional conformation in the *E*. *coli* system (**Fig 2A**).

Roseoflavin, lumiflavin and lumichrome inhibit riboflavin transport in some bacteria, *S*. *cerevisiae* and mammalian cells [12,13,16–18,21]. In this work, the three riboflavin analogs showed different effects on the parasites, with *T*. *cruzi* riboflavin uptake and proliferation most affected by roseoflavin (**Figs 1** and **S3**). The differences between the effects by the three analogs may be explained by their chemical structures (**Fig 1A and 1B, top panel**), where the high similarity between roseoflavin and riboflavin (only one substitution in the isoalloxazine ring) may enable this analog to mimic better the natural ligand. Additionally, roseoflavin presents antibiotic activity [65].

Human hepatocytes import roseoflavin and convert it by riboflavin kinase (EC 2.7.1.26) and FAD synthetase (EC 2.7.7.2) to Ro-FMN (roseoflavin mononucleotide) and Ro-FAD (roseoflavin adenine dinucleotide) analogs, and bind to intracellular flavoproteins, reducing or abolishing their function [46], impairing cell viability [65]. Recently, putative genes for riboflavin kinase (TcCLB.510741.80 and Tb09.211.3420) and FAD synthetase (TcCLB.508241.60) have been identified in *T*. *cruzi* and *T*. *brucei* [30,81]. Similarly to hepatocytes, trypanosomatids might import and convert roseoflavin to Ro-toxic analogs, impairing the flavin-related cellular metabolic processes and, ultimately, replication (**Figs 1B, S3** and **5F**). It is worth mentioning that flavoproteins has been proposed as targets for anti-infective strategies, reviewed in [82], including proteins related to the anti-oxidant systems dihydrolipoamide dehydrogenase (LipDH) and trypanothione reductase (TR). Several inhibitors have been successfully found for both flavoproteins, some of which show trypanocidal activity. In fact, LipDH is supposed to mediate at least partially the trypanocidal effect of nifurtimox and other nitrofurans [82]. Thus, effective *T*. *cruzi* riboflavin transport inhibition could eventually result in a depletion/reduction of its flavoenzyme pool, comprising LipDH and TR, and could lead–*per se* or in combination with other drugs–to effective parasite death.

We have shown that limiting availability to flavins affects metacyclogenesis (**Fig 5B and 5C**), a critical event in *T*. *cruzi* life cycle progression. Although the underlying molecular mechanisms in metacyclogenesis still remains unclear, antioxidants seem to be intimately involved in the epimastigote-MT cellular stage switch [70]. A proteomic analysis of metacyclogenesis has revealed increased levels of proteins related to anti-oxidant systems including LipDH [83]. It is possible that flavin restriction leads to a limited production of active flavoenzymes involved in anti-oxidant systems, and consequently to metacyclogenesis impairment as seen in **Fig 5B and 5C**.

To finish, it is noteworthy that *Tc*RibJ shows significant differences with the mammalian RFVT/SLC52 family: (i) they share very low sequence identity and similarity values (18.1–19.0% and 28,9–30,8%, respectively, **S5 Table**); (ii) only RibJ present MFS domains; (iii) they show a different number of predicted transmembrane segments (12 for *Tc*RibJ and 10–11 for RFVTs, **Fig 2A**) [12]; and (iv) RFVTs seem to be less sensitive to riboflavin derivatives and analogs than *Tc*RibJ as they required more than 200-fold excess concentration of such competitor compounds than the transporters of epimastigotes (see reference [84,85] and **Fig 1D and 1E**).

These differences, in addition to the results presented here that indicate the essentiality of riboflavin for *T*. *cruzi* survival and life cycle progression, pose *Tc*RibJ as a potential therapeutic target against Chagas disease.

## Supporting information

S1 Fig Trypanosomatid growth curves in control conditions. Parasites were grown until stationary phase, then washed and incubated in fresh SDM-20–10% FBS with the addition of 20 nM riboflavin. Cell density was quantified at days 3, 5 and 8.
(TIF)

S2 Fig Flavins promote the *in vitro* proliferation of trypanosomatids. Stationary phase trypanosomatids were washed and incubated in fresh SDM-20–10% FBS with the addition of different amounts of flavins (riboflavin: RF, FMN, or FAD). (A) *T*. *brucei* procyclic forms, (B) *L*. *(L*.*) mexicana* promastigotes, (C) *C*. *fasciculata* choanomastigotes and (D) *Phytomonas* Jma promastigotes were assayed. Trypanosomatid proliferation (%) was calculated counting parasites at the fifth day using control conditions (20 nM flavins) as reference (100%). Values are expressed as mean ± SD. Statistical analysis was performed by one way ANOVA test followed by a post-hoc Tukey's multiple comparison test (*P < 0.05, **P < 0.01, ***P < 0.005).
(TIF)

S3 Fig Riboflavin analogs inhibit *in vitro* proliferation of trypanosomatids. Parasites were maintained at 28°C in SDM-79 supplemented with 10% FBS. In the stationary phase, cells were washed with PBS and incubated in fresh SDM-79 supplemented with 10% FBS with the addition of analogs at 10 μM: (A) roseoflavin (RoF), (B) lumiflavin (LF) and (C) lumichrome (LC). Parasites were counted daily. Trypanosomatid proliferation (%) was calculated at the indicated round using fifth day-counts and using control condition without analog as reference (100%). Results obtained for *T*. *cruzi* were included for comparison. ND: differences not detected. Values are expressed as mean ± SD. Statistical analysis was performed by one way ANOVA test followed by a post-hoc Tukey's multiple comparison test (*P < 0.05, **P < 0.01, ***P < 0.005).
(TIF)

S4 Fig Riboflavin uptake apparent K_m_ and V_max_ parameters determinations in trypanosomatids. (A) *T*. *brucei* procyclic trypomastigotes, (B) *L*. *(L*.*) mexicana* promastigotes, (C) *C*. *fasciculata* choanamastigotes and (D) *Phytomonas* Jma promastigotes were used for the biochemical measurements. Parasites were grown in BHT media supplemented with FBS 10%, with the exception of *T*. *brucei* which was cultured in SDM-79 (FBS 10%), and cultured at 28°C until late log-phase. Cells were harvested, washed and resuspended in PBS- 2% glucose. The transport assays were performed in the range of 0–5 μM riboflavin (RF) final concentration. Aliquots were sampled at 0 and 5 min to calculate initial velocity. Values are expressed as mean ± SD. The apparent K_m_ and V_max_ values were obtained by nonlinear regression fit of the data to the Michaelis-Menten equation.
(TIF)

S5 Fig Maximum likelihood RibJ phylogenetic tree from Kinetoplastea. The tree was constructed using RibJ amino acid sequences from 37 trypanosomatids, 1 eubodonid and 1 parabodonid and Le and Gascuel model (-Ln = 8970.5397). Blue arrows indicate the RibJ of *T*. *cruzi*, *T*. *brucei* and *L*. *(L*.*) mexicana* studied during this work.
(TIF)

S6 Fig Heterologous expression of RibJ transporters does not alter membrane integrity. *E*. *coli* ∆*ribB* strain transformed with an empty vector or plasmids carrying *RibM*, *Tc*RibJ, *Tb*RibJ or *Lmi*RibJ were cultured in liquid LB with riboflavin excess at 37°C for 16 h with the addition of bactericidal compounds: (A) nalidixic acid (0, 2.5, 5, 10, 20 and 40 μg/mL), and (B) acriflavine (0, 10, 25, 50, 100 and 250 μg/mL). Values are expressed as mean ± SD.
(TIF)

S7 Fig Correlation analysis for maximum density and apparent K_m_ values for riboflavin uptake. The relative growth for each trypanosomatid (calculated as the maximum parasite count in riboflavin supplemented medium relative to control conditions) is plotted against its corresponding apparent K_m_ value. In this analysis, the relative growth obtained for the animal parasites (red) correlates with its transport properties (Pearson coefficient = 0.955, P < 0.05, represented as a grey area between the dotted lines). The plant parasite *Phytomonas* Jma (green) does not present this correlation.
(TIF)

S1 Table Primers designed to clone putative riboflavin transporters of *T*. *cruzi*, *T*. *brucei* and *L*. *(L*.*) mexicana*. (PDF)

S2 Table Identification of potential RibJ transporters in trypanosomatids with totally assembled genomes. (PDF)

S3 Table Identification of potential RibJ transporters in kinetoplastids with partially assembled genomes. (PDF)

S4 Table Amino acid sequence of riboflavin transporters used to construct the phylogenetic tree from Fig 3. (PDF)

S5 Table Comparison between human and *T. cruzi* riboflavin transporter sequences. (PDF)
