# Supplementary figures and images for: The superfamily keeps growing: Identification in trypanosomatids of RibJ, the first riboflavin transporter family in protists
Source: PLoS Negl Trop Dis. 2017 Apr 13;11(4):e0005513. doi: 10.1371/journal.pntd.0005513 (PMC5404878; doi:10.1371/journal.pntd.0005513)

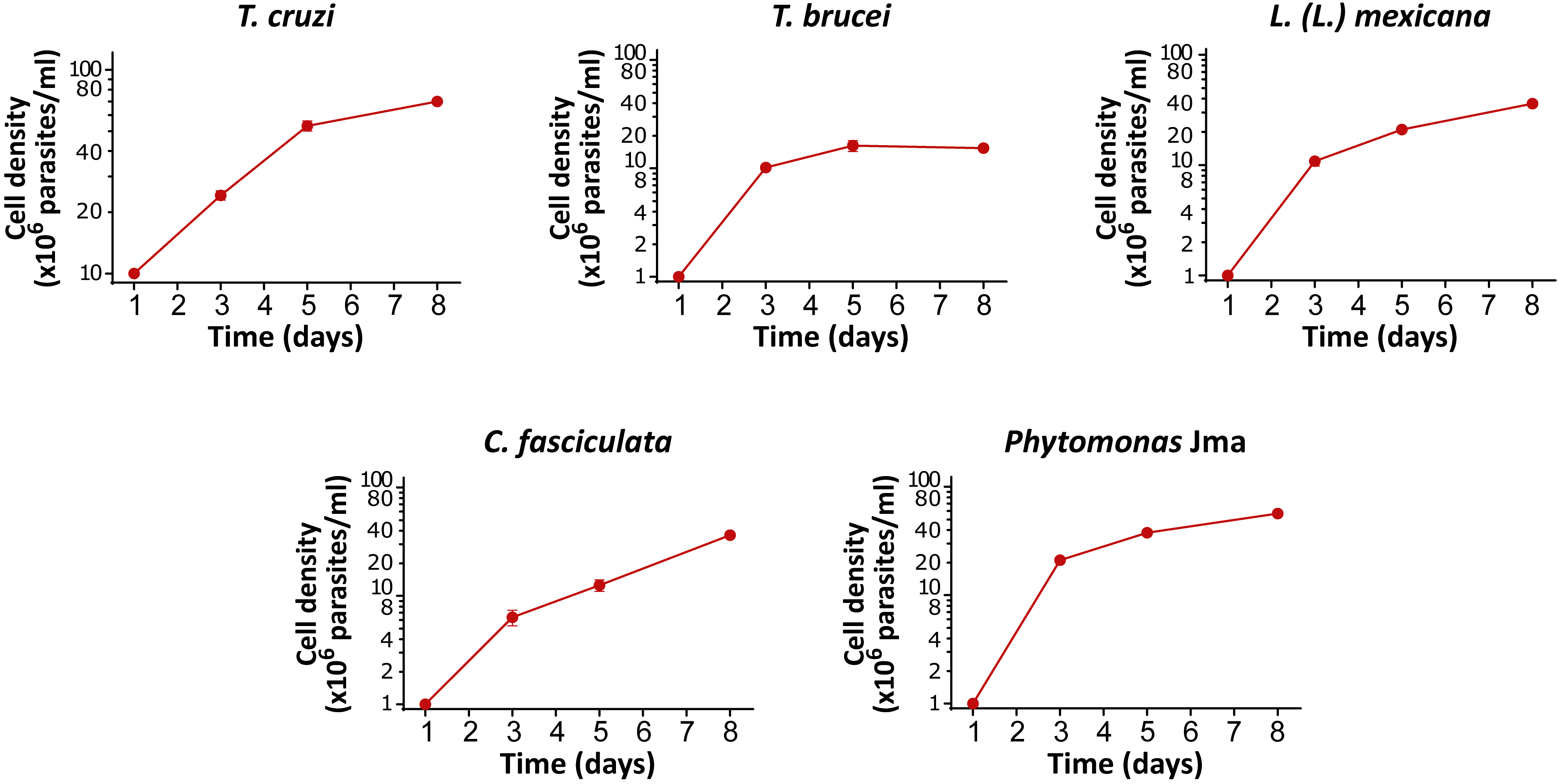

Supplement: S1 Fig — Parasites were grown until stationary phase, then washed and incubated in fresh SDM-20–10% FBS with the addition of 20 nM riboflavin. Cell density was quantified at days 3, 5 and 8. (TIF) [file pntd.0005513.s001.tif]

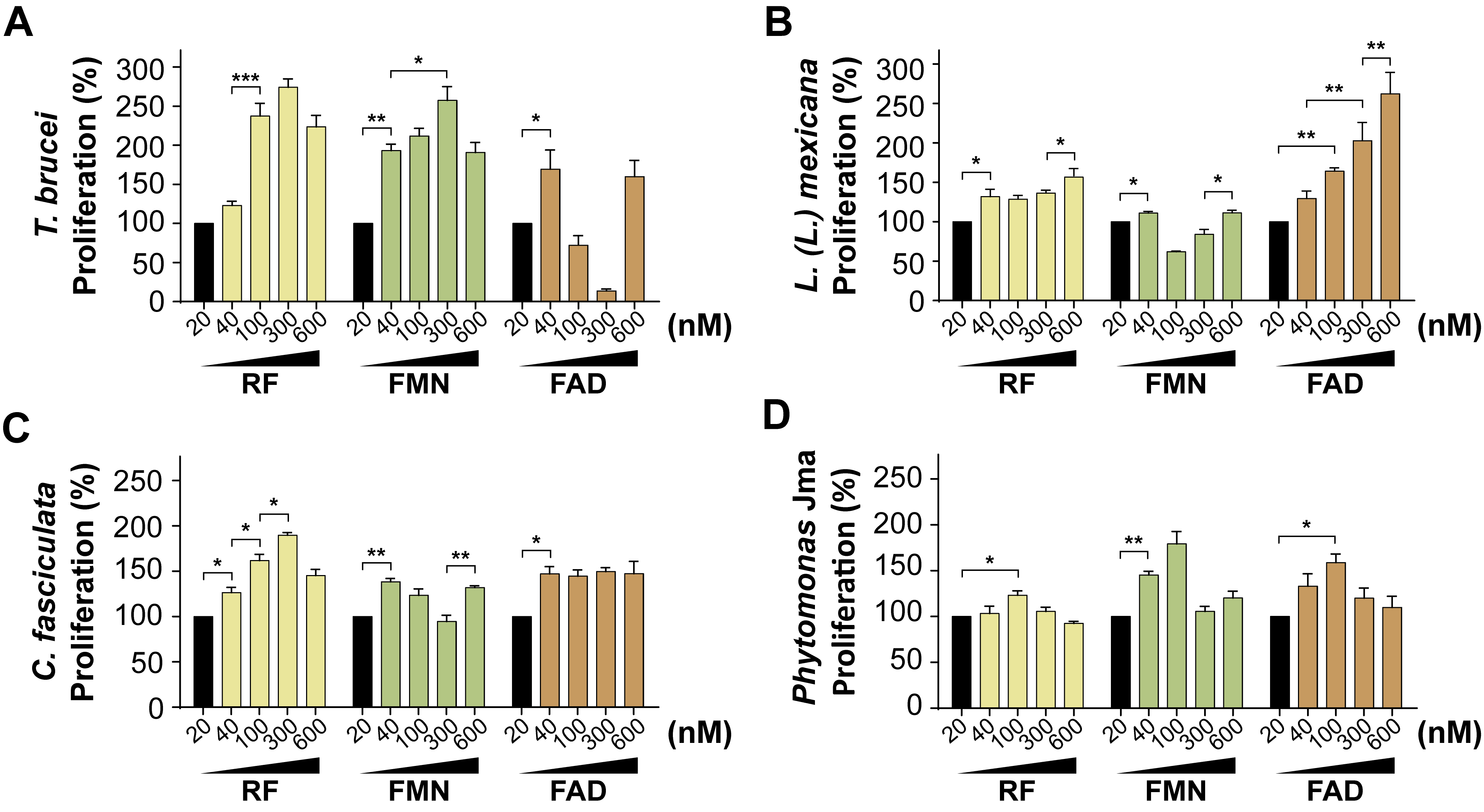

Supplement: S2 Fig — Stationary phase trypanosomatids were washed and incubated in fresh SDM-20–10% FBS with the addition of different amounts of flavins (riboflavin: RF, FMN, or FAD). (A) T. brucei procyclic forms, (B) L. (L.) mexicana promastigotes, (C) C. fasciculata choanomastigotes and (D) Phytomonas Jma promastigotes were assayed. Trypanosomatid proliferation (%) was calculated counting parasites at the fifth day using control conditions (20 nM flavins) as reference (100%). Values are expressed as mean ± SD. Statistical analysis was performed by one way ANOVA test followed by a post-hoc Tukey's multiple comparison test (*P < 0.05, **P < 0.01, ***P < 0.005). (TIF) [file pntd.0005513.s002.tif]

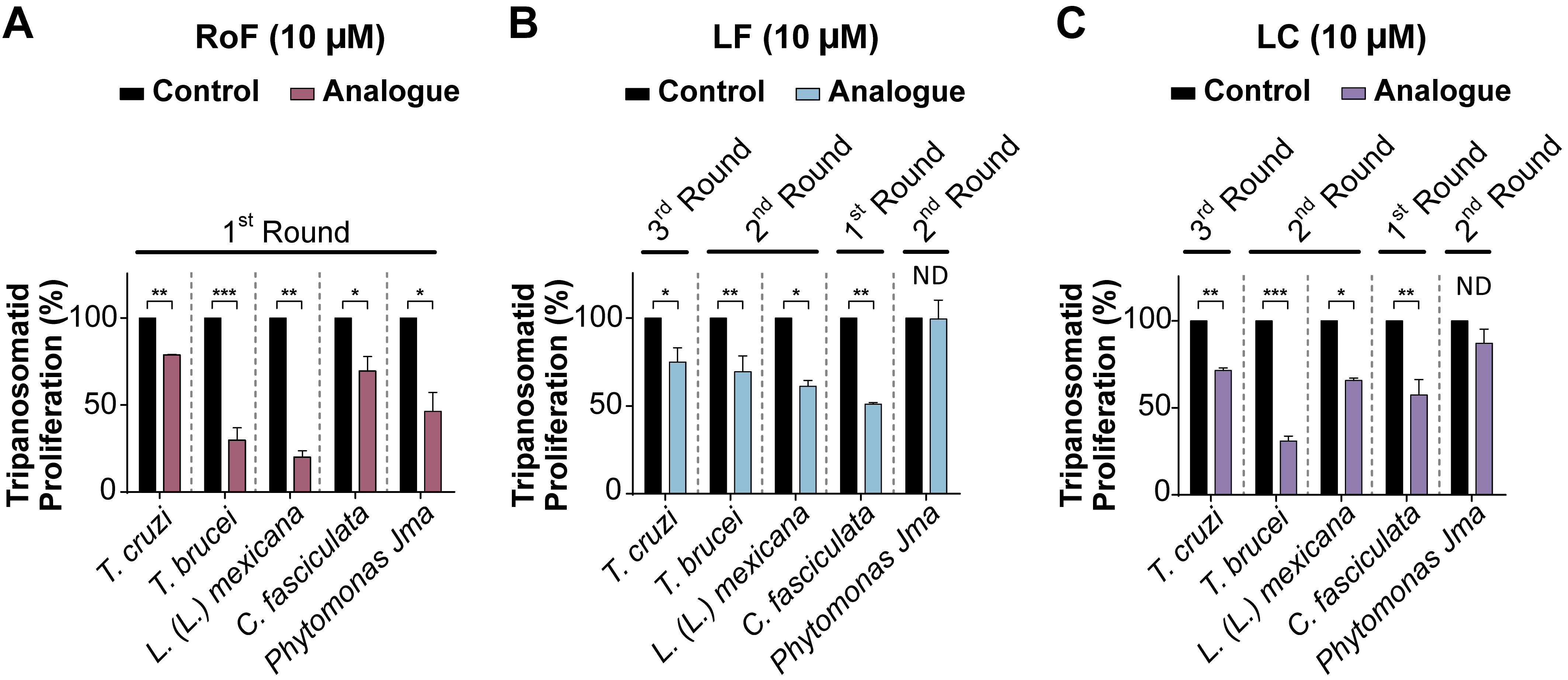

Supplement: S3 Fig — Parasites were maintained at 28°C in SDM-79 supplemented with 10% FBS. In the stationary phase, cells were washed with PBS and incubated in fresh SDM-79 supplemented with 10% FBS with the addition of analogs at 10 μM: (A) roseoflavin (RoF), (B) lumiflavin (LF) and (C) lumichrome (LC). Parasites were counted daily. Trypanosomatid proliferation (%) was calculated at the indicated round using fifth day-counts and using control condition without analog as reference (100%). Results obtained for T. cruzi were included for comparison. ND: differences not detected. Values are expressed as mean ± SD. Statistical analysis was performed by one way ANOVA test followed by a post-hoc Tukey's multiple comparison test (*P < 0.05, **P < 0.01, ***P < 0.005). (TIF) [file pntd.0005513.s003.tif]

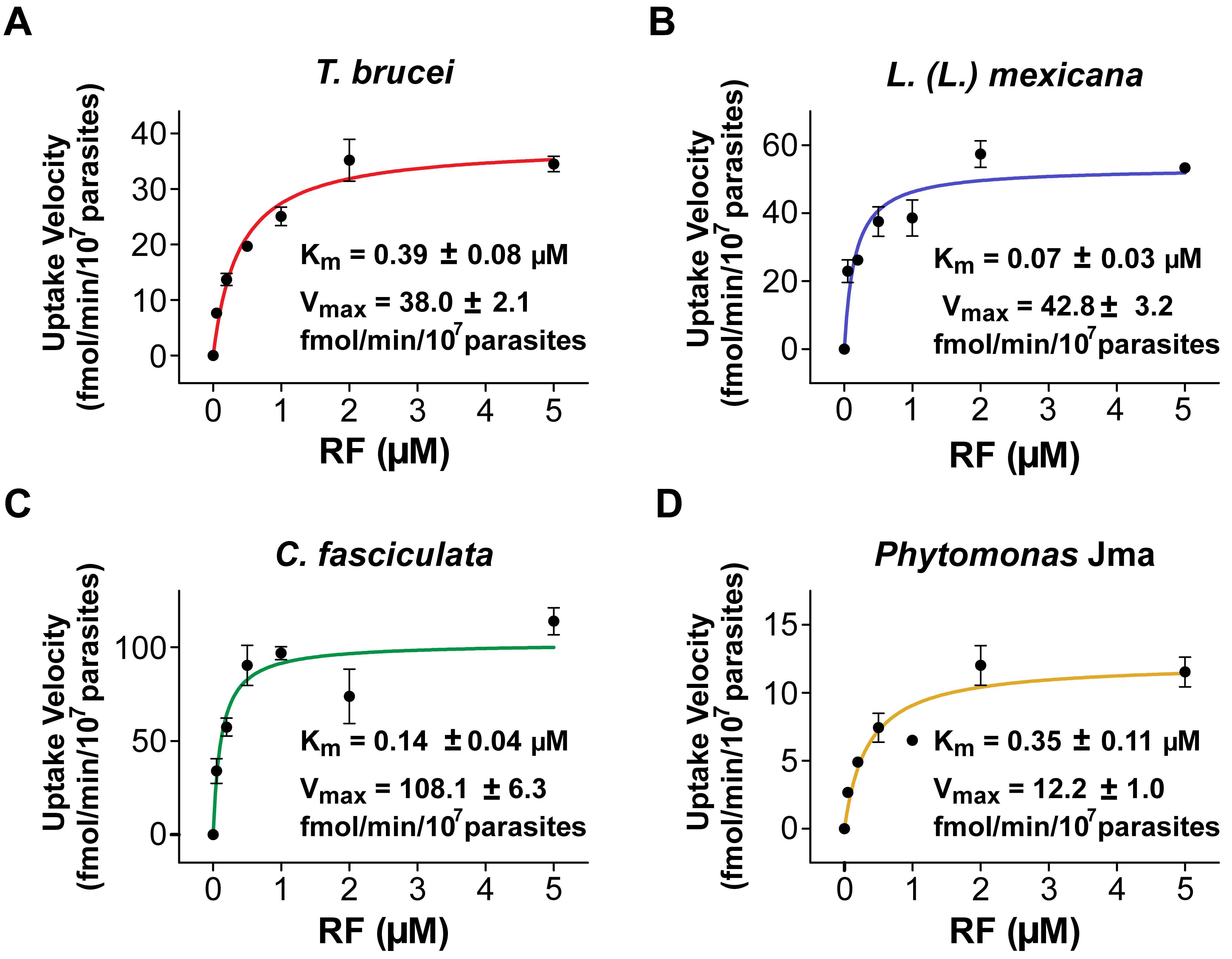

Supplement: S4 Fig — (A) T. brucei procyclic trypomastigotes, (B) L. (L.) mexicana promastigotes, (C) C. fasciculata choanamastigotes and (D) Phytomonas Jma promastigotes were used for the biochemical measurements. Parasites were grown in BHT media supplemented with FBS 10%, with the exception of T. brucei which was cultured in SDM-79 (FBS 10%), and cultured at 28°C until late log-phase. Cells were harvested, washed and resuspended in PBS- 2% glucose. The transport assays were performed in the range of 0–5 μM riboflavin (RF) final concentration. Aliquots were sampled at 0 and 5 min to calculate initial velocity. Values are expressed as mean ± SD. The apparent Km and Vmax values were obtained by nonlinear regression fit of the data to the Michaelis-Menten equation. (TIF) [file pntd.0005513.s004.tif]

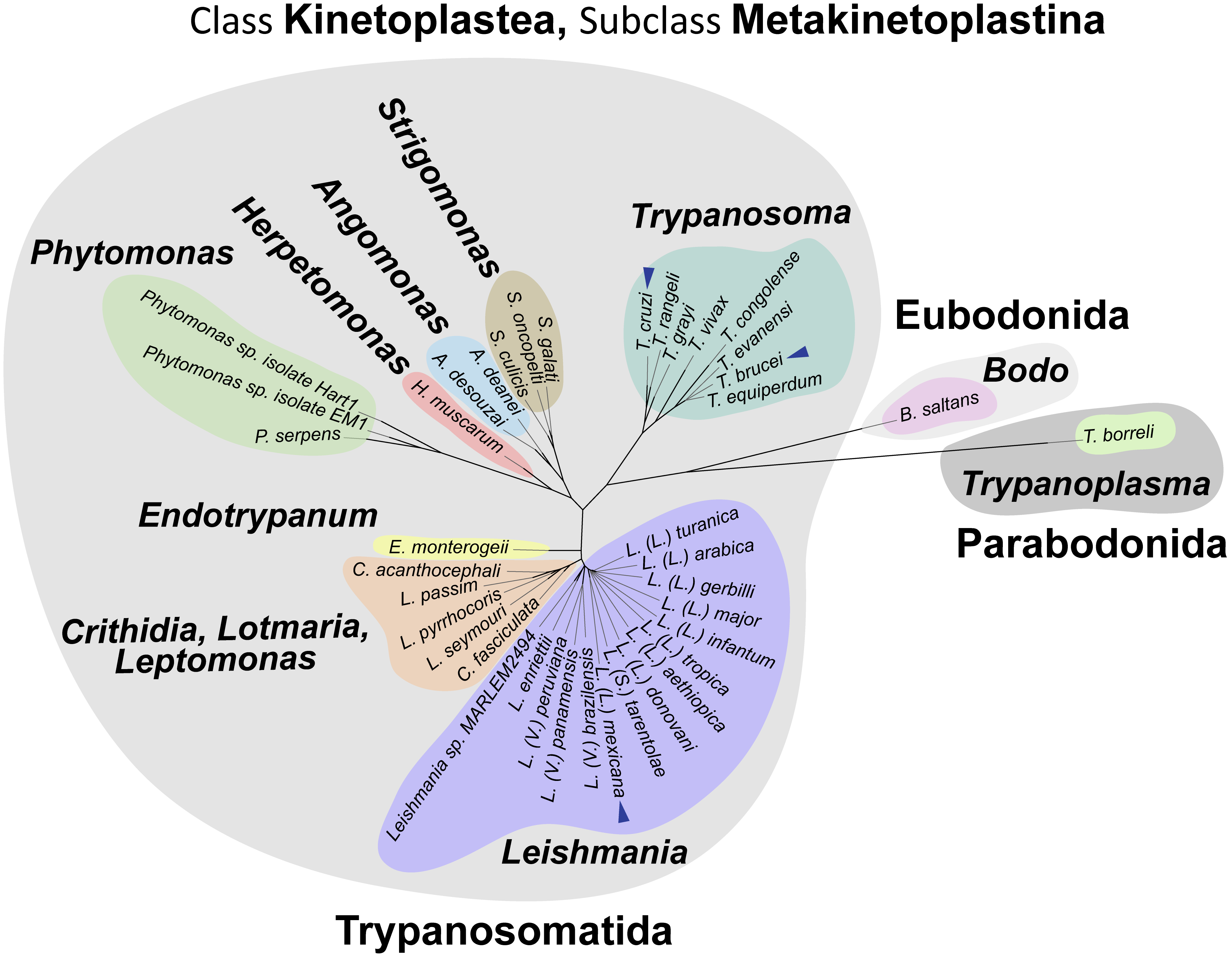

Supplement: S5 Fig — The tree was constructed using RibJ amino acid sequences from 37 trypanosomatids, 1 eubodonid and 1 parabodonid and Le and Gascuel model (-Ln = 8970.5397). Blue arrows indicate the RibJ of T. cruzi, T. brucei and L. (L.) mexicana studied during this work. (TIF) [file pntd.0005513.s005.tif]

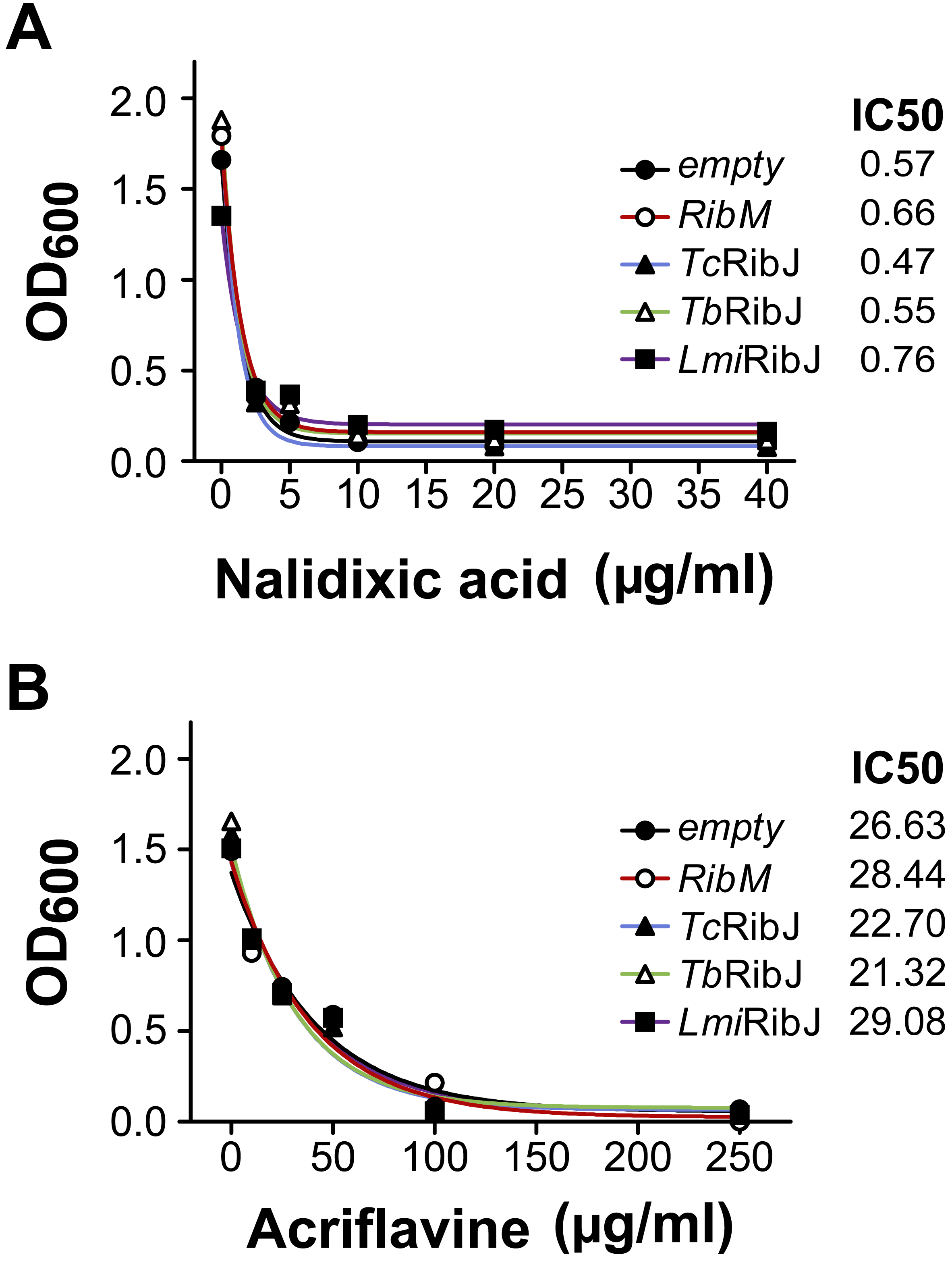

Supplement: S6 Fig — E. coli ∆ribB strain transformed with an empty vector or plasmids carrying RibM, TcRibJ, TbRibJ or LmiRibJ were cultured in liquid LB with riboflavin excess at 37°C for 16 h with the addition of bactericidal compounds: (A) nalidixic acid (0, 2.5, 5, 10, 20 and 40 μg/mL), and (B) acriflavine (0, 10, 25, 50, 100 and 250 μg/mL). Values are expressed as mean ± SD. (TIF) [file pntd.0005513.s006.tif]

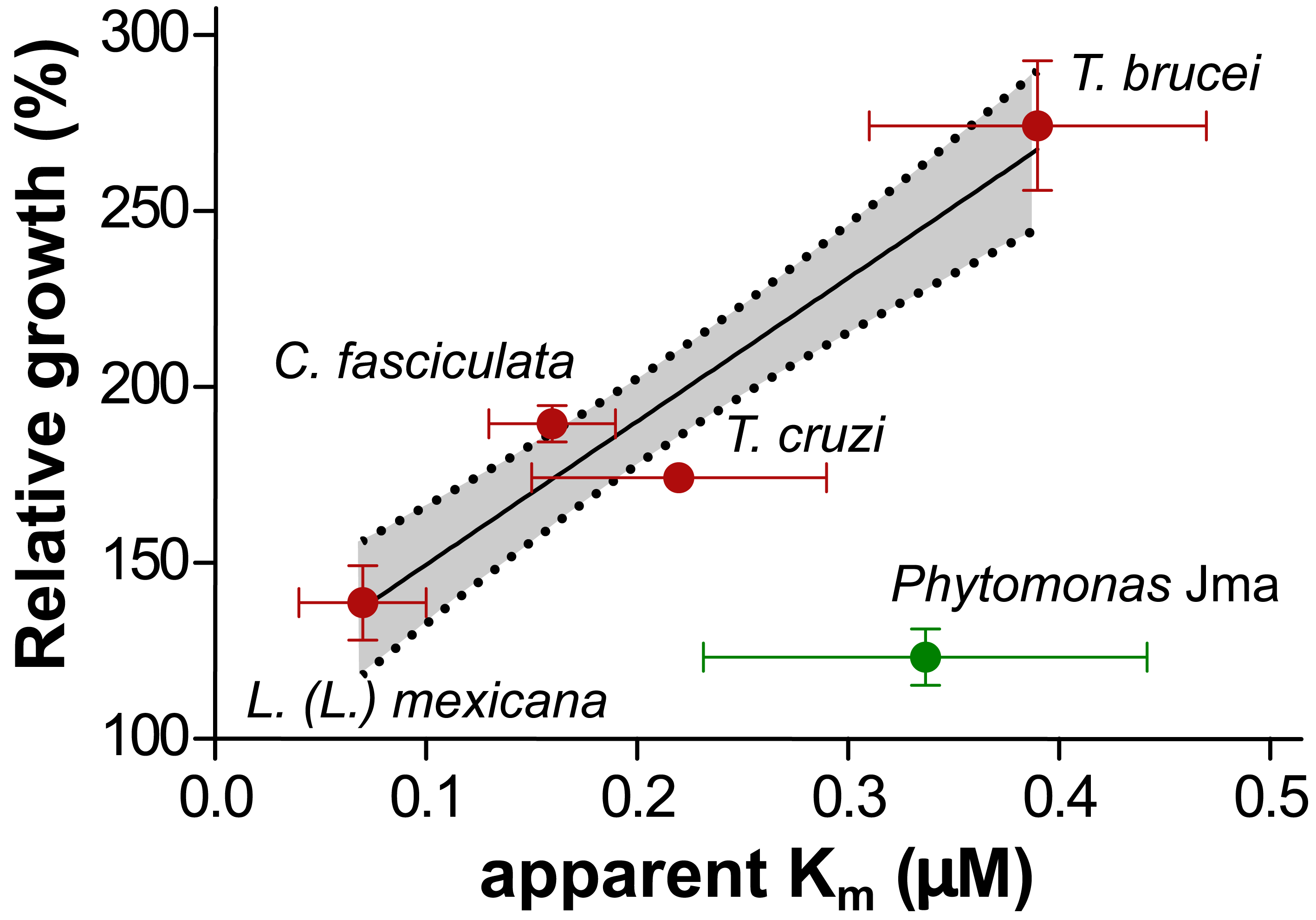

Supplement: S7 Fig — The relative growth for each trypanosomatid (calculated as the maximum parasite count in riboflavin supplemented medium relative to control conditions) is plotted against its corresponding apparent Km value. In this analysis, the relative growth obtained for the animal parasites (red) correlates with its transport properties (Pearson coefficient = 0.955, P < 0.05, represented as a grey area between the dotted lines). The plant parasite Phytomonas Jma (green) does not present this correlation. (TIF) [file pntd.0005513.s007.tif]
