# Supplementary material for: The superfamily keeps growing: Identification in trypanosomatids of RibJ, the first riboflavin transporter family in protists
Source: PLoS Negl Trop Dis. 2017 Apr 13;11(4):e0005513. doi: 10.1371/journal.pntd.0005513 (PMC5404878; doi:10.1371/journal.pntd.0005513)
Supplement: S1 Table — (PDF) [file pntd.0005513.s008.pdf]

S1 Table. Primers designed to clone putative riboflavin transporters of *T. cruzi, T. brucei* and *L. (L.) mexicana*.

**Primers**	**Sequence (5'- to 3'-)**
**F-EcoRI-*Tc*RibJ**	CGGAATTC**ATGTTGCCATGTTTCACACGG**
**R-HindIII-*Tc*RibJ**	CCCAAGCTT**TTACGCAAGTTTTGCTTCTGC**
**F-*Tc*RibJ-NdeI-6xHis**	TAACTTTAAGAAGGAGATATACATATG*CACCACCACCACCACCAC***TTGCCATGTTTCACACGGAAG**
**R-*Tc*RibJ-BamHI**	AGCTTGTCGACGGAGCTCGAATTCGGATCC**TTACGCAAGTTTTGCTTCTGC**
**F-*Tb*RibJ-NdeI-6xHis**	TAACTTTAAGAAGGAGATATACATATG*CACCACCACCACCACCAC***CTTCCAAGTTTCACCCGTAAA**
**R-*Tb*RibJ-BamHI**	AGCTTGTCGACGGAGCTCGAATTCGGATCC**TCACATAATCTCAACAAGTTT**
**F-*Lmi*RibJ-NdeI-6xHis**	TAACTTTAAGAAGGAGATATACATATG*CACCACCACCACCACCAC***CAGATCTACGAGGCATGCAAG**
**R-*Lmi*RibJ-BamHI**	AGCTTGTCGACGGAGCTCGAATTCGGATCC**TCACGCCTGCCTCACCACGCG**

Boldface: genomic sequences. Regular: vector sequences. Underlined: restrictions enzymes cleavage site. Italic: 6xHis tag sequence.
