# Supplementary material for: The superfamily keeps growing: Identification in trypanosomatids of RibJ, the first riboflavin transporter family in protists
Source: PLoS Negl Trop Dis. 2017 Apr 13;11(4):e0005513. doi: 10.1371/journal.pntd.0005513 (PMC5404878; doi:10.1371/journal.pntd.0005513)
Supplement: S2 Table — (PDF) [file pntd.0005513.s009.pdf]

S2 Table. Identification of potential RibJ transporters in trypanosomatids in totally assembled genomes.

	Parasite	Host	Accession number	MFS (e- value)[a]	Region[b]	*Tc*RibJ similarity (%)[c]
A	***Trypanosoma cruzi***	*Homo sapiens* (Chagas disease)	TcCLB.509885.70	Yes (1.7 e-16)	19-288	100
	Trypanosoma brucei	*Homo sapiens* (African trypanosomiasis)	Tb927.5.470	Yes (3.2 e-27)	19-337	62.0
	Leishmania (Leishmania) mexicana	*Homo sapiens* (cutaneous leishmaniasis)	LmxM.05.0480	Yes (7.2 e-19)	36-218	49.0
B	***Trypanosoma evansi***	*Bubalus bubalis* (water buffalo)	TevSTIB805.5.460	Yes (3.6 e-27)	19-377	62.5
	Trypanosoma congolense	*Bos* spp. (African bovine trypanosomiasis)	TcIL3000_0_01740	Yes (7.6 e-29)	19-354	60.5
	Trypanosoma grayi	*Glossina palpalis* (tsetse fly)	Tgr.1850.1000	Yes (9.4 e-24)	19-295	76.1
	Trypanosoma rangeli	*Homo sapiens* (asymptomatic)	TRSC58_01116	Yes (1.3 e-21)	19-288	83.3
	Trypanosoma vivax	*Bos taurus*	TvY486_0500070	Yes (8.0 e-27)	20-291	67.6
C	***Leishmania (Leishmania) tropica***	*Homo sapiens* (cutaneous leishmaniasis)	LTRL590_050009300	Yes (1.1 e-19)	39-218	49.8
	Leishmania (Leishmania) aethiopica	*Homo sapiens* (cutaneous leishmaniasis)	LAEL147_000064200	Yes (9.2 e-20)	39-218	49.8
	Leishmania (Viannia) braziliensis	*Homo sapiens* (cutaneous leishmaniasis)	LBRM2903_050010000	Yes (2.0 e-22)	33-375	50.9
	Leishmania (Viannia) panamensis	*Homo sapiens* (cutaneous leishmaniasis)	LPAL13_050009400	Yes (6.7 e-23)	33-373	50.8
	Leishmania sp. MAR LEM2494	*Homo sapiens* (cutaneous leishmaniasis)	LMARLEM2494_050009900	Yes (2.3 e-15)	33-392	48.7
	Leishmania (Leishmania) major	*Homo sapiens* (cutaneous leishmaniasis)	LmjF.05.0480	Yes (3.0 e-17)	39-393	49.8
	Leishmania (Leishmania) donovani	*Homo sapiens* (visceral leishmaniasis)	LdBPK_050480.1	Yes (8.7 e-16)	39-337	49.7
	Leishmania (Leishmania) infantum	*Homo sapiens* (infantile visceral leishmaniasis)	LinJ.05.0480	Yes (7.4 e-16)	39-337	49.7
	Leishmania (Leishmania) gerbelli	*Rhombomys opimus* (gerbil)	LGELEM452_050009800	Yes (1.2 e-16)	36-400	49.6
	Leishmania (Leishmania) turanica	*Rhombomys opimus* (gerbil)	LTULEM423_050009600	Yes (9.5 e-17)	39-396	50.0
	Leishmania (Leishmania) arabica	*Psammomys obesus* (rodent)	LARLEM1108_050009800	Yes (1.1 e-16)	36-396	49.5
	Leishmania (Sauroleishmania) tarentolae	*Tarentola mauritanica* (lizard)	LtaP05.0490	Yes (4.1 e-16)	33-383	49.2
	Leishmania enriettii complex	Rodents	ENLEM3045_050009900	Yes (1.2 e-20)	44-509	48.1
D	***Endotrypanum monterogeii***	*Choloepus hoffmanni* (sloth)	EMOLV88_050009300	Yes (3.3 e-22)	37-495	50.5
E	***Crithidia fasciculate***	*Anopheles quadrimaculatus* (mosquito)	CFAC1_020011500	Yes (1.2 e-15)	36-374	51.9
	Leptomonas pyrrhocoris	*Pyrrhocoris apterus* (european bug)	LpyrH10_07_0560	Yes (1.9 e-23)	36-364	52.6
	Leptomonas seymouri	*Dysdercus suturellus* (red bug)	Lsey_0441_0040	Yes (7.5 e-19)	33-480	52.4

Identification of putative riboflavin transporters using *Tc*RibJ as query in Tritryps database. [a]Predicted domains in Pfam server. [b]Regions included in the MFS domain. [c]Similarity with *Tc*RibJ obtained by ClustalW.

A: human parasites used in this work.
B: other parasites from the *Trypanosoma* genus.
C: other parasites from the *Leishmania* genus
D: other mammalian parasites.
E: parasites exclusive from insects.
