# Supplementary material for: The superfamily keeps growing: Identification in trypanosomatids of RibJ, the first riboflavin transporter family in protists
Source: PLoS Negl Trop Dis. 2017 Apr 13;11(4):e0005513. doi: 10.1371/journal.pntd.0005513 (PMC5404878; doi:10.1371/journal.pntd.0005513)
Supplement: S3 Table — (PDF) [file pntd.0005513.s010.pdf]

S3 Table. Identification of potential RibJ transporters in kinetoplastids with partially assembled genomes.

	Parasite	Host	Accession number (contig or scaffold)	MFS (e-value)[a]	Region[b]	*Tc*RibJ similarity (%)[c]	Reference
A	***Trypanosoma equiperdum***	Equines (venereal disease dourine)	CZPT01000402	Yes (3.2 e-27)	19-377	62.5	NP
B	***Leishmania (Viannia) peruviana***	*Homo sapiens* (visceral leishmaniasis)	LN609254.1	Yes (5.2 e-23)	33-375	50.8	[1]
C	***Angomonas desouzai***	*Ornidia obesa* (fly)	AUXL01001118	Yes (6.9 e-20)	35-309	55.4	[2]
	Angomonas deanei	*Zelus leucogrammus* (hemiptera)	AUXM01000876	Yes (1.3 e-24)	34-419	56.6	[2]
	Lotmaria passim	*Apis mellifera* (honey bee)	AHIJ01002325	Yes (1.4 e-16)	35-371	50.2	[3]
	Crithidia acanthocephali	*Acanthocephala femorata* (fly)	AUXI01000737	Yes (6.6 e-18)	36-370	51.5	[2]
	Herpetomonas muscarum	*Musca domestica* (fly)	AUXJ01001726	Yes (2.5 e-18)	38-355	49.8	[2]
	Strigomonas oncopelti	*Zelus leucogrammus* (hemiptera)	AUXK01003753	Yes (9.1 e-25)	33-474	50.4	[2]
	Strigomonas galati	-	AUXN01003492	Yes (7.6 e-18)	26-330	50.9	[2]
	Strigomonas culicis	*Aedes vexans* (mosquito)	AUXH01000639	Yes (8.1 e-19)	38-332	52.6	[2]
D	***Phytomonas serpens* 9T**	*Solanum lycopersicum* (isolate from tomato)	AIHY01002387	Yes (5.4 e-21)	74-412	38.1	[4]
	***Phytomonas sp.* isolate Hart1**	phloem-restricted pathogenic isolate from a diseased coconut from Guiana	HF955203 CAVR020000000*	Yes (1.7 e-22)	55-370	37.7	[5]
	***Phytomonas sp.* isolate EM1**	asymptomatic latex isolate from *Euphorbia*	HF955064 CAVQ010000000*	Yes (3.2 e-28)	61-429	39.9	[5]
E	***Bodo saltans***	None (free-living kinetoplastid)	BS47350	Yes (5.6 e-14)	12-413	39.9	[6]
	Trypanoplasma borreli	Cyprinid fish	NODE_24555_length_1382_cov_9.405933	Yes (6.0 e-24)	35-307	28.2	NP

Identification of putative riboflavin transporters using *Tc*RibJ as query in NCBI database. Currently, these genomes are partially assembled. [a]Predicted MSF domains by Pfam server. [b]Regions included in the MFS domain. [c]Similarity with *Tc*RibJ obtained by ClustalW. NP: Not published.

A: other parasites from the *Trypanosoma* genus.
B: other parasites from the *Leishmania* genus.
C: parasite exclusive from insects.
D: phytoparasites.
E: parasite belonging to different order than Trypanosomatida.

Supplementary References

1. Valdivia HO, Reis-Cunha JL, Rodrigues-Luiz GF, Baptista RP, Baldeviano GC, Gerbasi R V., et al. Comparative genomic analysis of Leishmania (Viannia) peruviana and Leishmania (Viannia) braziliensis. BMC Genomics. BMC Genomics; 2015;16: 715. doi:10.1186/s12864-015-1928-z
2. Alves JMP, Klein CC, da Silva FM, Costa-Martins AG, Serrano MG, Buck GA, et al. Endosymbiosis in trypanosomatids: the genomic cooperation between bacterium and host in the synthesis of essential amino acids is heavily influenced by multiple horizontal gene transfers. BMC Evol Biol. 2013;13: 190. doi:10.1186/1471-2148-13-190
3. Runckel C, DeRisi J, Flenniken ML. A Draft Genome of the Honey Bee Trypanosomatid Parasite Crithidia mellificae. PLoS One. 2014;9: e95057. doi:10.1371/journal.pone.0095057
4. Koreny L, Sobotka R, Kovarova J, Gnipova A, Flegontov P, Horvath A, et al. Aerobic kinetoplastid flagellate Phytomonas does not require heme for viability. Proc Natl Acad Sci U S A. 2012;109: 3808–3813. doi:10.1073/pnas.1201089109/-/DCSupplemental.www.pnas.org/cgi/doi/10.1073/pnas.1201089109
5. Porcel BM, Denoeud F, Opperdoes F, Noel B, Madoui M-AA, Hammarton TC, et al. The Streamlined Genome of Phytomonas spp. Relative to Human Pathogenic Kinetoplastids Reveals a Parasite Tailored for Plants. PLoS Genet. 2014;10: e1004007. doi:10.1371/journal.pgen.1004007
6. Jackson AP, Quail M a, Berriman M. Insights into the genome sequence of a free-living Kinetoplastid: Bodo saltans (Kinetoplastida: Euglenozoa). BMC Genomics. 2008;9: 594. doi:10.1186/1471-2164-9-594
