# Supplementary material for: The superfamily keeps growing: Identification in trypanosomatids of RibJ, the first riboflavin transporter family in protists
Source: PLoS Negl Trop Dis. 2017 Apr 13;11(4):e0005513. doi: 10.1371/journal.pntd.0005513 (PMC5404878; doi:10.1371/journal.pntd.0005513)
Supplement: S4 Table — (PDF) [file pntd.0005513.s011.pdf]

S4 Table. Amino acid sequence of riboflavin transporters used to construct the phylogenetic tree from Fig. 2B.

Classification	Species	Name	Accession number	Reference
**Mammals**	*Homo sapiens* RFVT1	RFVT/SCL52	Q9NWF4.2	[1]
	Homo sapiens RFVT3		Q9NQ40.4	[1]
	Homo sapiens RFVT2		Q9HAB3.1	[1]
	Mus musculus RFVT2		Q9D8F3.1	[2]
	Pan paniscus member 1		XP_003810223.1	Predicted
	Pongo abelii member 2		XP_002833179.2	Predicted
	Macaca fascicularis member 2		XP_005564405.1	Predicted
	Macaca nemestrina member 1		XP_011724203.1	Predicted
	Equus caballus member 1		XP_014583611.1	Predicted
	Tursiops truncates member 2		XP_004319077.1	Predicted
	Orcinus orca member 2		XP_012386875.1	Predicted
	Bos mutus member 2		XP_014334325.1	Predicted
	Ictidomys tridecemlineatus member 2		XP_005316095.1	Predicted
	Cricetulus griseus member 2		XP_003507685.1	Predicted
	Equus przewalskii member 2		XP_008508436.1	Predicted
	Ceratotherium simum simum member		XP_004443067.1	Predicted
	Ratus norvegicus member 3		NP_001032275.1	Predicted
	Myotis brandtii member 3		XP_014402039.1	Predicted
	Gorilla gorilla gorilla member 3		XP_004061693.1	Predicted
	Pan troglodytes member 3		XP_009434927.1	Predicted
	Macaca mulatta member 2		NP_001181490.1	Predicted
	Pantholops hodgsonii member 3		XP_005981822.1	Predicted
**Fungi**	*Saccharomyces cerevisiae*	MCH5	NP_014951.4	[3]
	Arthroderma otae		XP_002843476.1	Predicted
	Colletotrichum fioriniae		XP_007594293.1	Predicted
	Colletotrichum gloeosporioides		XP_007279280.1	Predicted
	Eutypa lata		XP_007792122.1	Predicted
	Fibroporia radiculosa		XP_012184704.1	Predicted
	Gaeumannomyces graminis		XP_009221625.1	Predicted
	Magnaporthe oryzae		XP_003716530.1	Predicted
	Marssonina brunnea		XP_007296567.1	Predicted
	Microsporum gypseum		XP_003169176.1	Predicted
	Ogataea parapolymorpha		XP_013933620.1	Predicted
	Paracoccidioides lutzii		XP_002797527.1	Predicted
	Verticillium alfalfa		XP_003000372.1	Predicted
	Verticillium dahlia		XP_009648470.1	Predicted
	Wickerhamomyces ciferrii		XP_011271917.1	Predicted
**Nematodes**	*Caenorhabditis elegans*	rft	CCD69385.2	[4]
	Brugia malayi		XP_001896372.1	Predicted
	Loa loa		XP_003145255.1	Predicted
	Necator americanus		XP_013300104.1	Predicted
	Trichinella spiralis		XP_003380128.1	Predicted

The sequences were obtained from the NCBI Protein database annotated as predicted riboflavin transporters. Function has not been established for the entries with the exception of transporters belonging to *H. sapiens*, *M. musculus*, *C. elegans* and *S. cerevisiae*.

Supplementary References

1. Yonezawa A, Inui KI. Novel riboflavin transporter family RFVT/SLC52: Identification, nomenclature, functional characterization and genetic diseases of RFVT/SLC52. Mol Aspects Med. 2013;34: 693–701. doi:10.1016/j.mam.2012.07.014
2. Yao Y, Yonezawa A, Yoshimatsu H, Omura T, Masuda S, Matsubara K. Involvement of riboflavin transporter RFVT2/Slc52a2 in hepatic homeostasis of riboflavin in mice. Eur J Pharmacol. 2013;714: 281–7. doi:10.1016/j.ejphar.2013.07.042
3. Reihl P, Stolz J. The monocarboxylate transporter homolog Mch5p catalyzes riboflavin (vitamin B2) uptake in Saccharomyces cerevisiae. J Biol Chem. 2005;280: 39809–39817. doi:10.1074/jbc.M505002200
4. Biswas A, Elmatari D, Rothman J, LaMunyon CW, Said HM. Identification and functional characterization of the Caenorhabditis elegans riboflavin transporters rft-1 and rft-2. PLoS One. 2013;8: e58190. doi:10.1371/journal.pone.0058190
