# Supplementary material for: The superfamily keeps growing: Identification in trypanosomatids of RibJ, the first riboflavin transporter family in protists
Source: PLoS Negl Trop Dis. 2017 Apr 13;11(4):e0005513. doi: 10.1371/journal.pntd.0005513 (PMC5404878; doi:10.1371/journal.pntd.0005513)
Supplement: S5 Table — (PDF) [file pntd.0005513.s012.pdf]

S5 Table. Comparison between human and *T. cruzi* riboflavin transporter sequences.

		H. sapiens					
		**RFVT1 (Q9NWF4.2)**		**RFVT2 (Q9HAB3.1)**		**RFVT3 (Q9NQ40.4)**	
		**% identity**	**% similarity**	**% identity**	**% similarity**	**% identity**	**% similarity**
T. cruzi	**TcRibJ (TcCLB.509885.70)**	18,5	28,5	19,0	30,9	18,1	30,1
	**TcRibJ (TcCLB.508397.70)**	18,9	28,9	19,5	30,8	18,4	29,3
